# Evolutionary history and postglacial colonization of an Asian pit viper (*Gloydius halys caucasicus*) into Transcaucasia revealed by phylogenetic and phylogeographic analyses

**DOI:** 10.1038/s41598-018-37558-8

**Published:** 2019-02-04

**Authors:** Atefeh Asadi, Claudine Montgelard, Masoud Nazarizadeh, Akram Moghaddasi, Faezeh Fatemizadeh, Evgeniy Simonov, Haji Gholi Kami, Mohammad Kaboli

**Affiliations:** 1 CEFE, PSL-EPHE (Biogéographie et Ecologie des Vertébrés), CNRS, University Montpellier, Univ Paul Valéry Montpellier 3, IRD, Montpellier, France; 2 Department of Environmental Science, Faculty of Natural Resources, University of Tehran, Karaj, Iran; 3 Laboratory of Forest Genomics Siberian Federal University, 660036 Akademgorodok 50a/2, rasnoyarsk, Russia; 4 Department of Biology, Faculty of Sciences, Golestan University, Gorgan, Iran; 5 Centre for Ecological Genomics and Wildlife Conservation, Department of Zoology, University of Johannesburg, Johannesburg, South Africa

## Abstract

It has been generally acknowledged that glacial climates at the time of the Pleistocene altered the patterns of species distributions, prompting latitudinal and altitudinal distribution shifts in several species, including poikilothermic species commonly known for their thermal sensitivity. However, the historical phylogeographic patterns of such species have remained largely unknown. Here, we present the historical biogeographic, phylogenetic, and phylogeographic relationships of the Caucasian pit viper, *G*. *h*. *caucasicus*, based on two mtDNA (cyt *b* and ND4) and one nDNA (c-mos) genes. This pit viper represents the westernmost member of the Crotalinae subfamily in the Palearctic and occurs in a variety of habitats, from 30 m to 3,000 m above sea level. In Iran, it is distributed on the northern and southern slopes of the Alborz Mountains, rendering it a target for phylogenetic and phylogeographic studies of a terrestrial poikilothermic animal. Our study identified four Iranian lineages of *G*. *h*. *caucasicus* along the northeastern to northwestern slopes of the Alborz Mountains and southern Azerbaijan (Talysh Mountains). Diversification of the Iranian lineages highlights population expansion and subsequent isolation into four plausible refugial areas during the Quaternary paleo-climatic oscillations, confirmed by our molecular dating and historical biogeographic analyses. The results of coalescence-based simulations support the incursion of the species from northeastern Iran to the western end of the Alborz, and then toward Transcaucasia via two directions: northern and southern slopes of the Alborz Mountains. Furthermore, our results clearly implied that *G*. *h*. *caucasicus* should be elevated to species rank and further referred to as *G*. *caucasicus* (Nikolsky, 1916).

## Introduction

It has been widely accepted that different glacial events during the Pleistocene have shaped the current phylogeographical structure and distribution of taxa throughout northern temperate regions^1–4^. Many studies have highlighted that lineages currently occupying the Old World had formerly inhabited areas which were later covered by ice sheets during glaciation events. As glaciers advanced, populations were driven toward southern glacial refugia^5,6^, where they subsequently dispersed into newly available habitats during interglacial periods^2,7^.

Iran is a mountainous country with more than half of its mainland covered by mountains^8^. The Alborz encompasses a series of mountain ranges extending from northwestern to northeastern Iran^9^. The formation of the Alborz Mountains was first initiated during the Paleocene and the rugged landscape of the Alborz took shape during the early Cenozoic. This uplift was the outcome of an expansive movement throughout Iran as well as the Caucasian Mountains^10,11^. In addition, the Hyrcanian forests along the northern slopes of the Alborz Mountains and southern coasts of the Caspian Sea rank among the most important forest remnants in western Eurasia and are characterized by having one of the most ecologically valuable biodiversity hotspots in the Middle East^8^.

Recent research indicates that continental glaciers in Iran during the Pleistocene^12,13^, as well as the current climate change in northern Iran^14^, could have, respectively, led to latitudinal and altitudinal distribution shifts in a number of species, particularly poikilothermic animals, which are noted for their sensitivity to changes in temperature^15^. The Caucasian pit viper *Gloydius halys caucasicus* (Nikolsky, 1916) is distributed throughout southeastern Azerbaijan, southern Turkmenistan (Kopet Dagh Mountains), from northeastern to northwestern Iran, and northwestern Afghanistan, and is relatively common across the Alborz Mountains. This viper is a member of the *G*. *halys/G*. *intermedius* species complex, which represents a group of closely related vipers of the Crotalinae subfamily (Viperidae), including a total of nine taxa: *G*. *halys halys*, *G*. *h*. *caucasicus*, *G*. *caraganus*, *G*. *cognatus*, *G*. *stejnegeri*, *G*. *rickmersi*, *G*. *shedaoensis*, *G*. *changdaoensis*, and *G*. *intermedius*^16–19^. With a widespread range in the Palearctic, they inhabit a spectrum of various biotopes distributed across an extensive territory from Azerbaijan and Iran through several countries of Central Asia to eastern Siberia, Mongolia, and China^17,20–22^. Although this complex has been the focus of numerous phylogenetic^16,17,20,23–28^, morphological^24,29^, ecological^30,31^, and captive-breeding studies^32^, it remains an enigmatic species group. The intricacy arises out of a recent discovery of a morphologically and genetically distinct species^17^, evincing the fact that the diversity within this complex is most likely underestimated. Additionally, Wagner *et al*.^17^ proposed the elevation of the Caucasian pit viper from subspecies to species rank. This was later accepted by Shi *et al*. (2016, 2017). Moreover, Shi *et al*.^19^ argued for elevation of two other subspecies *G*. *h*. *cognatus* and *G*. *h*. *stejnegeri*, to the full species rank, which was later reaffirmed by Shi *et al*.^18^.

The Caucasian pit viper occupies a diverse range of habitat types, from 30 m to about 3,000 m above sea level, within northern and southern slopes of the Alborz Mountains, thus serving as an ideal example to evaluate phylogenetic and phylogeographic patterns of a terrestrial poikilothermic species in northern Iran. Even though this species has been under intensive exploitation for venom milking by the Razi Vaccine and Serum Research Institute since 1924^26^, the details of its evolutionary history and population structure have remained poorly understood to this day^17,33,34^.

Developments in molecular taxonomy and barcoding techniques allow rapid detection of cryptic diversity^35^. It has been demonstrated that combining molecular, morphological, ecological, and biological data is a crucial key to detecting cryptic species, especially in less well-known areas. Moreover, the growing concern toward conservation of genetic diversity calls for accurately defining evolutionary significant units (ESUs) based on evolutionary histories of relevant taxa^36^ (see^37^ for a review of ESU definitions), as species-based units cannot always prove applicable to all conservation strategies^38^. Thus, phylogenetic and phylogeographic inferences are applied to conservation planning below the species level^13,39^.

In this study, we sought to better understand the phylogeny, phylogeography, and taxonomic reassessment of *G*. *h*. *caucasicus* across its entire distribution range in Iran and Azerbaijan, using partial mtDNA sequences of the Cytochrome *b* (cyt *b*) and NADH dehydrogenase subunit 4 (ND4) genes, as well as the nuclear proto oncogene c-mos. We (i) delineated the entire geographically defined evolutionary lineages of this viper and their spatial distribution from northeastern to northwestern Iran and southern Azerbaijan. We (ii) performed coalescent simulations to compare several historical biogeographical hypotheses (single refugium or multiple refugia, along with one-way or two-way gene flows) that involve lineage diversification within this viper. Then, we (iii) used statistical phylogenetic methods to evaluate the taxonomic status of this subspecies within the *G*. *halys*/*G*. *intermedius* species complex. Finally, (iv) we used our analyses to uncover patterns of historical migration of the species to Transcaucasia and to define ESUs of this viper. Given the genetic structure and distribution of the Iranian lineages, we proposed some recommendations for effective conservation of all phylogenetically significant lineages of the Caucasian pit viper in Iran.

## Results

### Phylogenetic reconstruction

In total, 1618 aligned positions including cyt *b* and ND4 were analyzed with 1332 invariable, 36 singleton, and 250 parsimony-informative sites, along with a total of 48 haplotypes, 24 of which belonged to *G*. *h*. *caucasicus*. However, the highly conserved nuclear c-mos fragment with a sequence length of 567 bp showed only 563 invariable, three singleton, one parsimony-informative sites, and a total of three haplotypes. No insertions, deletions, or stop codons were detected. Furthermore, base composition was estimated T = 26.7%, C = 32.2%, A = 30.0%, and G = 11.1% for mtDNA genes, and T = 29.4%, C = 19.2%, A = 29.8%, and G = 21.5% for nDNA gene. Based on HKA tests, the levels of DNA polymorphism in our mtDNA dataset conformed to expectations of neutral evolution. HKA tests were non-significant for the ND4 (N = 84 ingroup sequences, χ2 = 0.015, *P* = *0*.*901*) and the cyt *b* (N = 88 ingroup sequences, χ2 = 0.001, *P* = *0*.*973*) datasets. Moreover, The PHI test revealed no statistically significant proof for nuclear recombination *P* = *0*.*207*.

**Figure 1:**
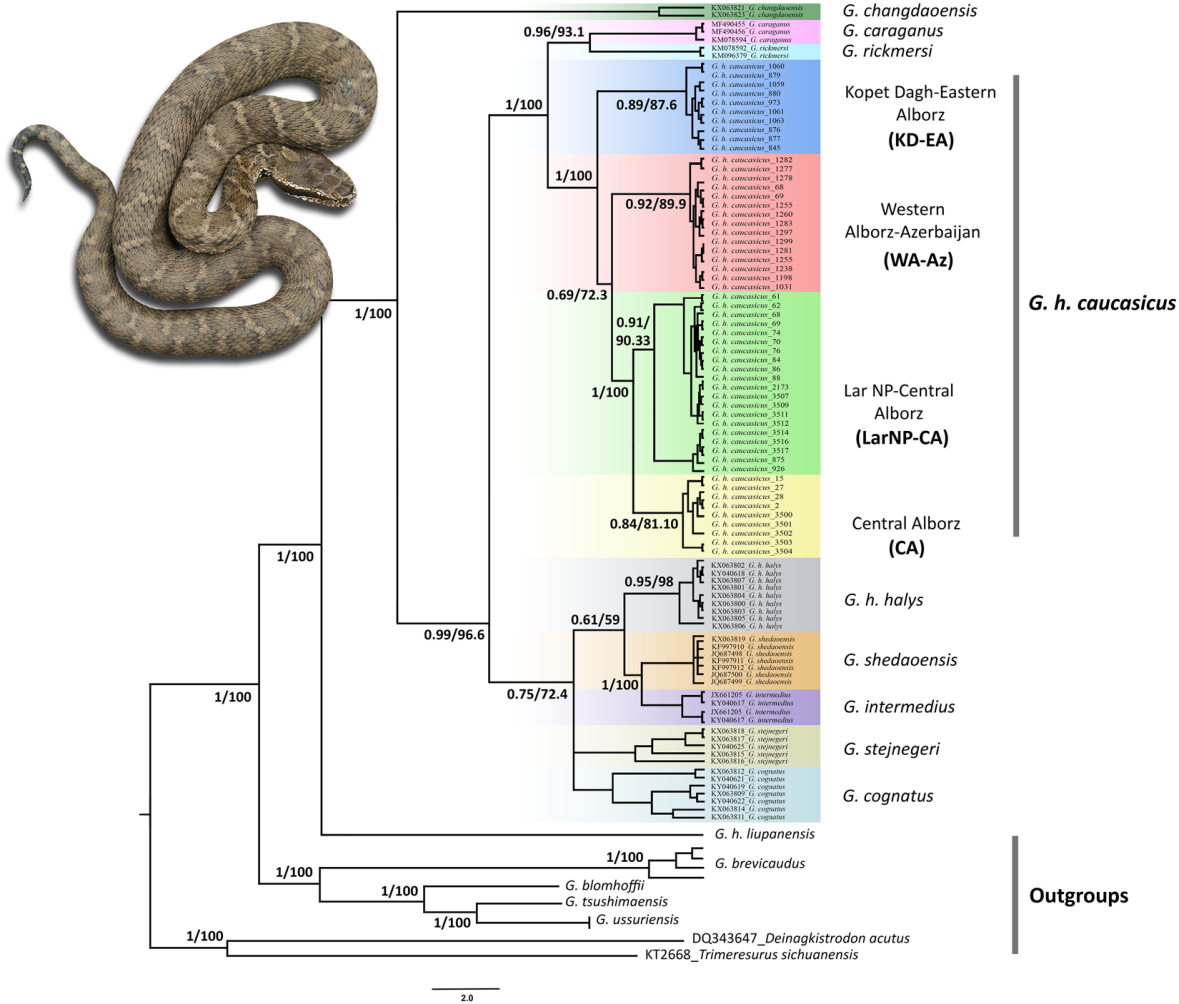
Bayesian 50% majority-rule consensus tree reconstructed from concatenated mtDNA (cyt *b* + ND4) + nDNA (c-mos) dataset (in congruence with ML tree in terms of the branching pattern and the positions of the clades), using six outgroups (*G*. *brevicaudus*, *G*. *blomhoffi*, *G*. *tsushimaensis*, *G*. *ussuriensis*, *D*. *acutus*, and *T*. *sichuaensis*). Nodal support presented at nodes indicate Bayesian posterior probability (left) by MrBayes, and ML bootstrap (right) using IQ-TREE.

PartitionFinder found three subset partitions for the three genes; (i) ND4-pos1/cyt *b*-pos2/c-mos-pos1, (ii) ND4-pos2/cyt *b*-pos3/c-mos-pos2, and (iii) ND4-pos3/cyt *b*-pos1/c-mos-pos3 with the best-fitting models of nucleotide substitution TRN + G, HKY + G, and HKY + I, respectively. This partitioning strategy was used for both the Bayesian Inference (BI) and Maximum Likelihood (ML) analyses. The resulting phylogenetic trees obtained by both methods were congruent in the branching pattern (Fig. 1). The basal divergence of *G*. *changadaoensis* from the remaining species of the complex was well supported (BI posterior probability = 1 and ML bootstrap value = 100). In addition, east Asian (*G*. *h*. *halys*, *G*. *cognatus*, *G*. *stejnegeri*, *G*. *intermedius*, and *G*. *shedaoensis*) and west Asian (*G*. *h*. *caucasicus*, *G*. *caraganus*, and *G*. *rickmersi*) vipers of the complex formed well-differentiated clades supported by high values of posterior probability (0.99) and bootstrap support (96.6). All lineages of *G*. *h*. *caucasicus* constitute a monophyletic group that is positioned as a sister clade to *G*. *rickmersi* and *G*. *caraganus* with high support (1.0 posterior probability and 100 bootstrap support). Moreover, the Kopet Dagh-Eastern Alborz (KD-EA) lineage formed a strongly well-supported clade with the other three Caucasian clades. However, the Western Alborz-Azerbaijan (WA-Az) lineage formed a separate clade with moderate bootstrap (72.3) and posterior probability (0.69) support, whereas the Lar National Park-Central Alborz (LarNP-CA) lineage and the Central Alborz (CA) separated from one another with high posterior probability (1) and bootstrap (100) support.

Furthermore, all samples of *G*. *h*. *halys* formed a well-supported clade, which, together with the remaining taxa (*G*. *cognatus*, *G*. *stejnegeri*, *G*. *intermedius*, and *G*. *shedaoensis*) emerged as a moderately supported clade (posterior probability of 0.75 and bootstrap support of 72.4). All in all, 12 clades were identified for the *G*. *halys*/*G*. *intermedius* complex, consisting of eight well-supported (KD-EA, LarNP-CA, and WA-Az lineages of *G*. *h*. *caucasicus*, along with *G*. *intermedius*, *G*. *shedaoensis*, *G*. *rickmersi*, *G*. *caraganus* and *G*. *changadaoensis*) and four moderately-supported (CA lineage of *G*. *h*. *caucasicus*, *G*. *h*. *halys*, *G*. *cognatus*, and *G*. *stejnegeri*) clades (Fig. 1).

### Bayesian species delimitation and genetic distances among the *G*. *halys/G*. *intermedius* complex species

Our BPP analysis with algorithm 1 and under three different scenarios of prior values revealed that the different prior values for $\theta$ and τ0 did not alter the posterior speciation probabilities. Based on the results of our first scenario, each of the nine delimited taxa corresponding to *G*. *halys halys*, *G*. *h*. *caucasicus*, *G*. *caraganus*, *G*. *cognatus*, *G*. *stejnegeri*, *G*. *rickmersi*, *G*. *shedaoensis*, *G*. *changdaoensis*, and *G*. *intermedius* were strongly supported with 95% Bayesian posterior probability (PP = 1), considering three models with small and large ancestral sizes, as well as deep and shallow divergences. The genetic distances among *G*. *changdaoensis*, *G*. *intermedius*, *G*. *shedaoensis*, *G*. *cognatus*, *G*. *stejnegeri*, and *G*. *caraganus* vary from 1.0%–5.4% (the blue cells in Table S3), whereas the average genetic distance between the four Iranian lineages of *G*. *h*. *caucasicus* and the other taxa in the complex varies from 3.7–5.6% (the grey cells in Table S3), which is comparable to that among valid species members of this complex.

According to our second scenario, we did not obtain high Bayesian posterior probabilities for the four Iranian clades of *G*. *h*. *caucasicus* (0.93–95%) in our BPP analysis, under the three models with small and large ancestral sizes, as well as deep and shallow divergences. Moreover, the genetic distances among the Iranian lineages including the two clades of Central Alborz (LarNP-CA and CA), the eastern clade (KD-EA), and the western clade (WE-Az) vary from 2.4–2.9% (the green cells in Table S3); however, the distance drops to 0.15% between the two clades of Central Alborz (LarNP-CA and CA), in concordance with node support values of BI and ML gene trees.

### Haplotype network

**Figure 2:**
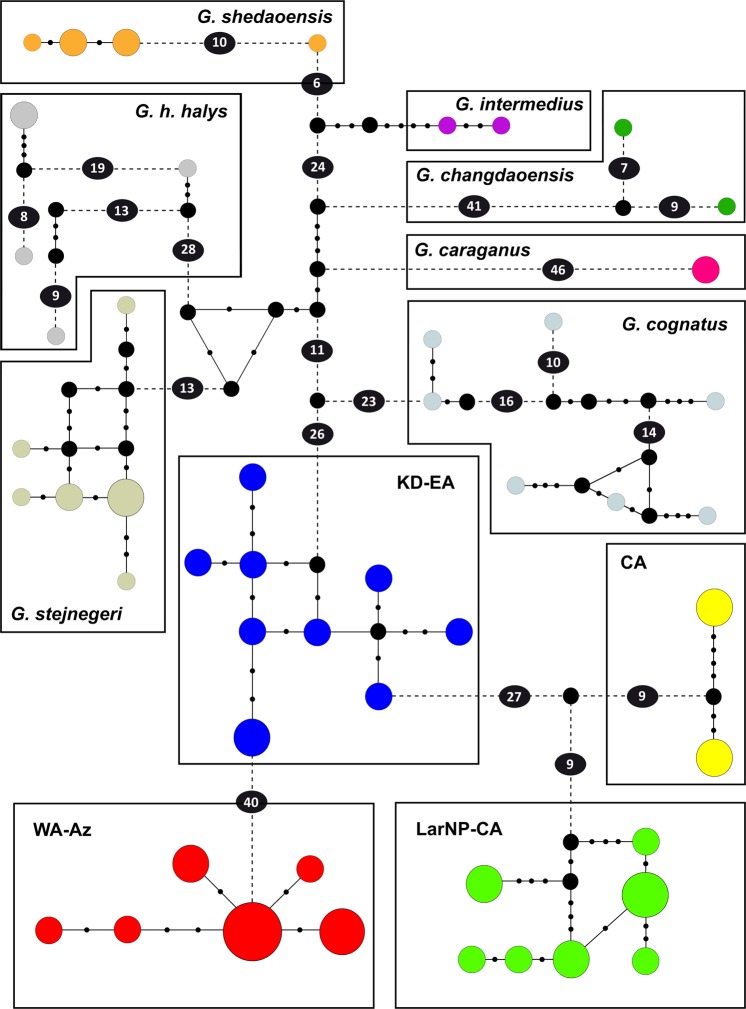
Haplotype network using the 1618 bp of the concatenated sequences from cyt *b* + ND4 and a total of 70 sequences. Statistical parsimony network assuming a 95% parsimony threshold, as constructed by TCS. Symbol size and branch lengths are proportional to the number of shared individuals per haplotype and the number of mutational steps among haplotypes, respectively. Black circles indicate unsampled or extinct haplotypes. Sizes of circles are proportional to haplotype frequencies. Small black circles indicate one mutational step.

Haplotypes plotted with TCS v1.21 on our mtDNA matrix (with 39 samples of *G*. *h*. *caucasicus* and 31 samples of the other taxa of *G*. *halys/G*. *intermedius* complex) revealed a significant divergence within the complex, in which *G*. *h*. *caucasicus* was distant from *G*. *cognatus* (49 mutational steps), *G*. *stejnegeri* (52 mutational steps), *G*. *h*. *halys* (67 mutational steps), *G*. *caraganus* (84 mutational steps), *G*. *changdaoensis* (82 mutational steps), *G*. *intermedius* (65 mutational steps) and *G*. *shedaoensis* (71 mutational steps) (Fig. 2). Within *G*. *h*. *caucasicus*, in accordance with the phylogenetic tree, we found four significant clusters from northeastern Iran to western Alborz-Azerbaijan. These four clusters were separated from each other by 18–40 mutational steps. Most central haplotypes in LarNP-CA, CA, and WA-Az clusters were shared by 2–5 individuals, while within the KD-EA cluster, haplotypes mostly corresponded to single individuals. The WA-Az cluster included six closely related haplotypes comprising the most western known locality for the complex in the western Palearctic.

Maximum and minimum nucleotide diversity was attributed to CA and LarNP-CA lineages, respectively. Additionally, haplotype diversity ranged from 0.667 to 0.978 in WA-Az and KD-EA lineages, respectively (Table 1).

**Table 1:** Number of haplotypes (*p*), nucleotide diversity (*pi*), haplotype diversity (*h*), and number of polymorphic sites (*H*) of the Caucasian pit viper lineages.

Lineages	n	*p*	*pi*	*h*	*H*
*G*. *h*. *caucasicus*	39	24	0.02049	0.964	24
Kopet Dagh-Eastern Alborz (KD-EA)	10	12	0.0020	0.978	9
Central Alborz (CA)	4	6	0.00247	0.667	2
LarNP-Central Alborz (LarNP-CA)	12	11	0.00186	0.894	7
Western Alborz-Azerbaijan (WA-Az)	13	6	0.00194	0.679	6

### Spatial genetic structure of *G*. *h*. *caucasicus* lineages in northern Iran and Azerbaijan

Using the concatenated mtDNA + nDNA dataset, the BAPS results divided the samples into the four clusters concordant with the phylogenetic tree and haplotype network; (i) KD-EA including Hezar Masjid Mountains, Khorasan, and Golestan provinces, (ii) LarNP-CA comprising eastern and central parts of Mazandaran province as well as Lar National Park, (iii) CA containing western Mazandaran and eastern Gilan provinces, and (iv) WA-Az including western Gilan and Ardebil provinces, as well as the southern mountains of Azerbaijan. Additionally, AMOVA on our concatenated mtDNA dataset proved a high rate of variation (89.38%) among the four clusters, and the fixation index (FST) confirmed a significant genetic structure among the clusters (Table S1).

### Divergence time

**Figure 3:**
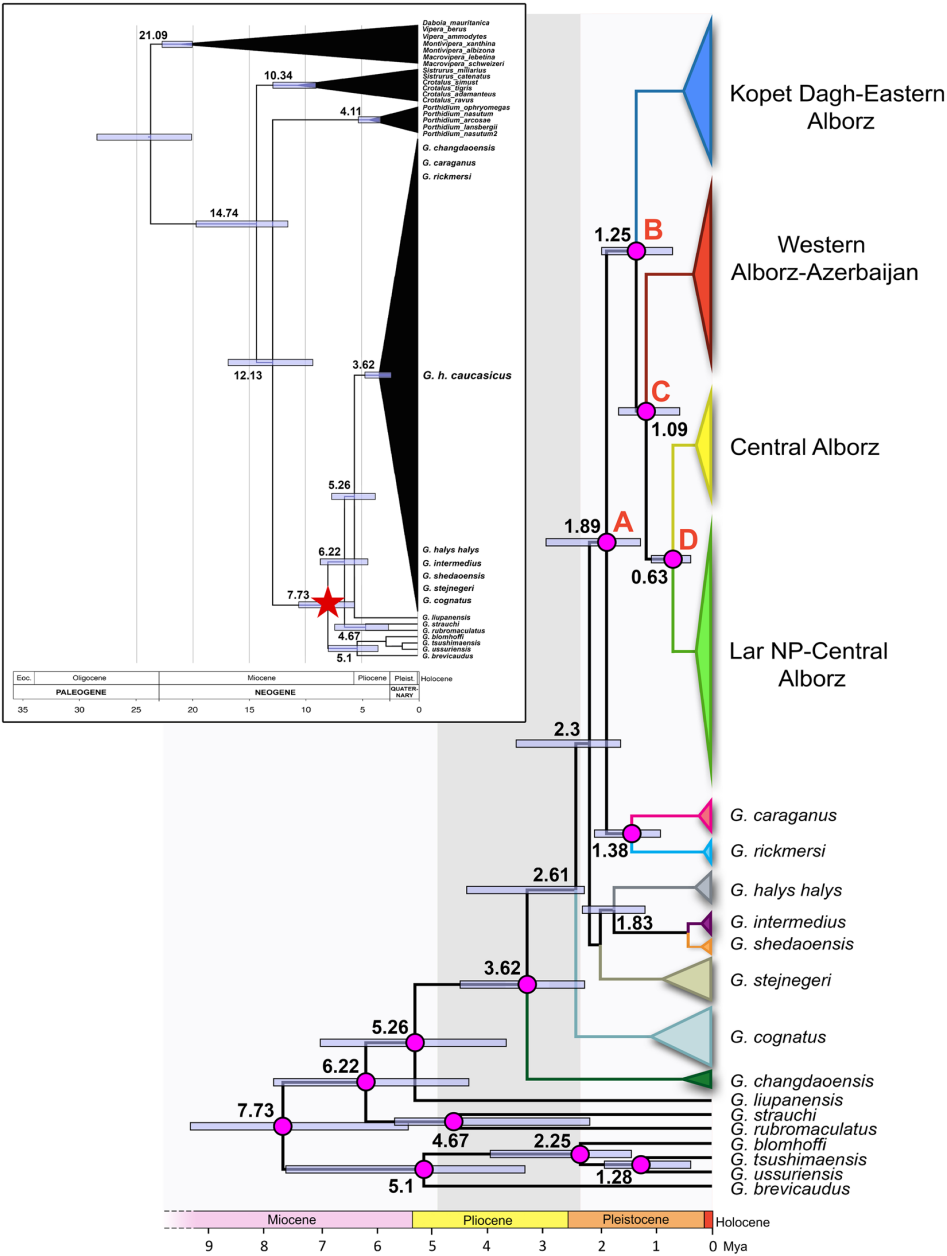
Chronogram of the *G*. *halys/G*. *intermedius* complex based on the concatenated mtDNA + nDNA dataset and a total of 114 sequences (89 *Gloydius* and 25 outgroups). Divergence times were estimated using a Lognormal clock and a Birth-Death Process model. The black chronogram inset on the top left corner displays dating of the entire dataset and the red star denotes the starting point of the evolutionary relationships shown by the phylogeny on the right. The pink circles represent nodes with posterior probability PP > 90%.

Our molecular clock dating based on concatenated mtDNA + nDNA dataset revealed that *G*. *h*. *caucasicus* separated from its sister clade (*G*. *caraganus* and *G*. *rickmersi*) in the early Pleistocene (1.89 Myr, 95% HPD: 1.20–2.70 Myr; node A, Fig. 3), while divergence within *G*. *h*. *caucasicus* commenced in the mid-Pleistocene (1.25 Myr, 95% HPD: 0.73–1.83 Myr), for which three main diversifications can be defined. The first period of diversification corresponds to the separation of the KD-EA from northeastern Iran and the other lineages (node B, Fig. 3) in the Calabrian stage. The second split between CA and WA-Az lineages (node C, Fig. 3) occurred at 1.09 Myr (95% HPD: 0.64–1.64 Myr) during the same stage as node B. The last divergence occurred at the center of Alborz where two lineages (CA and LarNP-CA) diverged from each other at 0.68 Myr (95% HPD: 0.34–0.89 Myr, node D in Fig. 3) during the mid-Pleistocene (Ionian stage).

### Historical biogeography at the Palearctic scale

**Figure 4:**
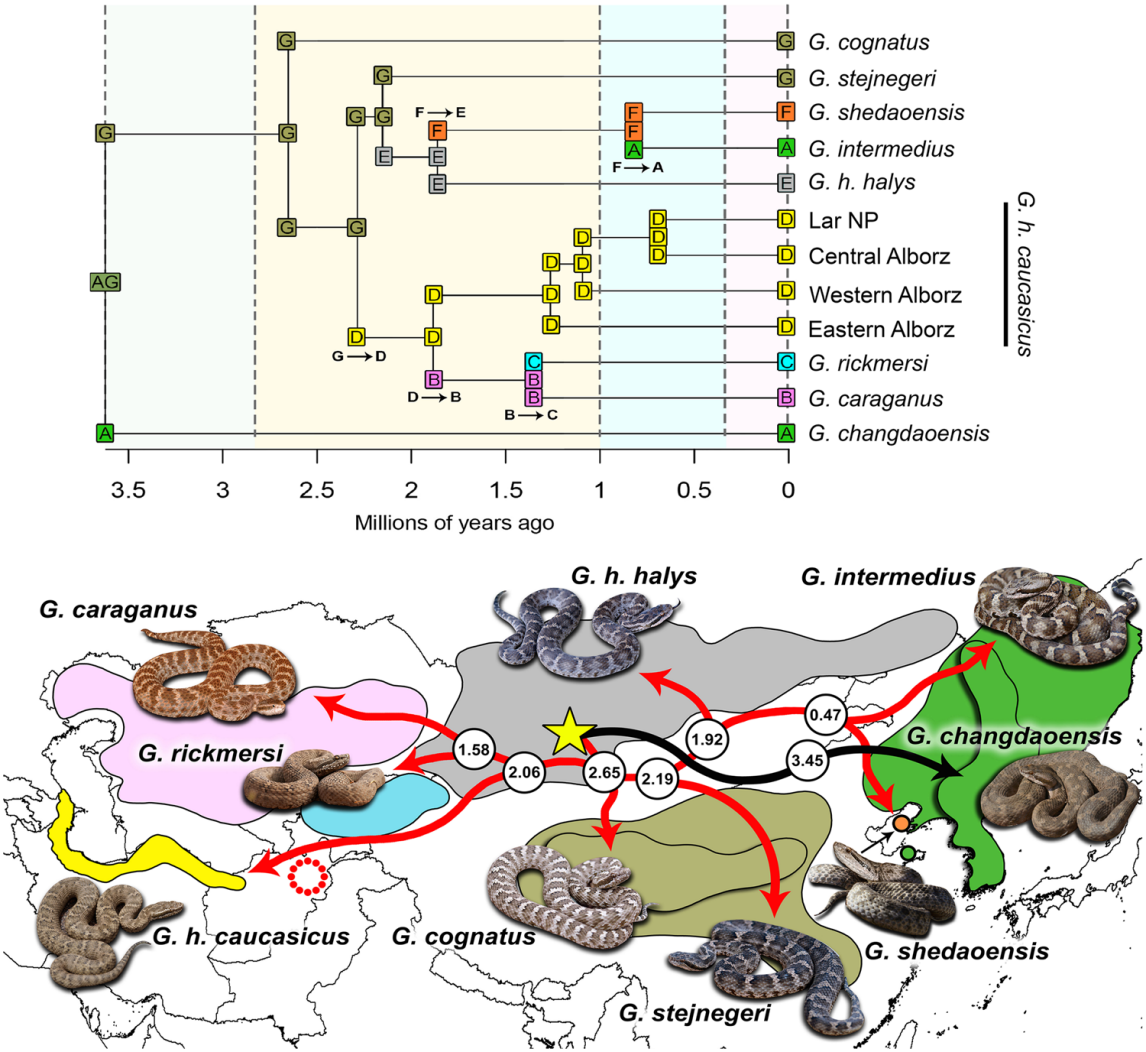
Ancestral range estimation for the *G*. *halys/G*. *intermedius* complex using BioGeoBEARS under DIVALIKE + J model on the concatenated mtDNA + nDNA dataset. Colours are correspondent to the seven regions (explained in section 2.4.2). Yellow star indicates ML estimation of the center of origin of all taxa (latitude 43.308 and longitude 96.388, estimated by PhyloMapper 1b1) in the complex, the orange circle shows *G*. *shedaoensis* locality in Shedao Island, China, and black and red arrows present a hypothetical direction of dispersal supported by BioGeoBEAR results. Numbers refer to divergence times among taxa in million years (see Fig. 3). Polygons show approximate range of distribution of each species/subspecies. The red circle in Afghanistan refers to *G*. *h*. *boehmei* (Nilson, 1983), which is not included in our study.

Based on the Akaike information criterion, the historical biogeography analysis using the concatenated mtDNA + nDNA dataset revealed that the DIVALIKE + J model holds the strongest support compared to other analyses performed in BioGeoBEARS (Table S2) and dispersal, extinction, and cladogenesis parameters were estimated at 0.000431, 0.022, and 1.00E-12, respectively. We have found some allopatric speciation and founder‐event speciation in evolution of this complex in different time slices (Fig. 4). Furthermore, among the four different scenarios tested, the second one (Scenario_HB_ in Table S2) is characterized by the highest AICc value, under the DIVALIKE + J model (Fig. 4). The best hypothesis suggests that the ancestor of the complex was widespread through regions A and G at 3.62 (2.30–5.32) Mya, which went extinct at 2.61 (1.72–3.69) Mya from region A. Regions D and E were colonized from region G by the ancestor of *G*. *stejnegeri* and *G*. *cognatus* at 2.30 (1.52–3.22) Mya and 2.15 (1.44–3.06) Mya, respectively. In addition, regions B and F were separately colonized from regions D and E by the ancestors of *G*. *h*. *caucasicus* and *G*. *h*. *halys* at 1.89 (1.20–2.70) Mya and 1.83 (1.08–2.68) Mya.

### Coalescent analyses and simulations of *G*. *h*. *caucasicus*

Both ABC coalescence simulations (direct and the logistic approaches) using the concatenated mtDNA + nDNA dataset led to the conclusion that the Scenario_EH_ 3 is the best supported scenario (Pp = 0.844) compared to the other hypotheses (Pp = 0.458 and 0.411). Furthermore, model choice validation using PODS indicates that adequate power exists for selecting the true hypothesis among competing hypotheses with an acceptable Type I error rate (35%) and low Type II error rate (16%). These results hint at the incursion of the species from northeastern Iran to the western end of the Alborz and then toward the southern mountains of Azerbaijan (Talysh Mountains) through two directions, via northern and southern slopes of the Alborz Mountains. Furthermore, our results may suggest that the observed regional phylogeographic pattern may likely reflect colonization into four allopatric refugia during the Pleistocene glaciation episodes.

### Species Distribution Modelling

**Figure 5:**
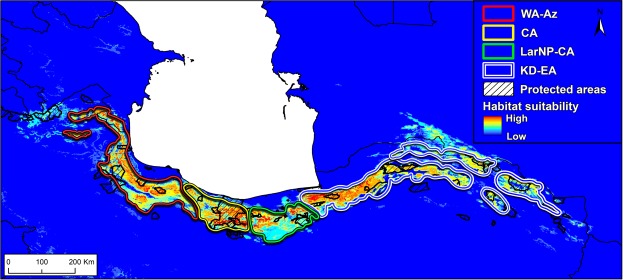
Habitat suitability map of *G*. *h*. *caucasicus* populations in northern Iran. Coloured polygons represent areas occupied by each lineage. Crosshatch polygons show the network of protected areas of Iran and its overlap with the suitability map. Boundaries of lineages were detected by BAPS.

Our SDM based on the Biomod framework showed high average values of discrimination capacity (AUC = 0.93–0.97) and classification accuracy (TSS = 0.89–0.92) for the four modelling methods. The final ensemble model illustrates that the most suitable landscape for *G*. *h*. *caucasicus* is the southern and northern slopes of the Alborz Mountains (Fig. 5). The variables of proximity to forest cover, roughness, maximum temperature of the warmest month, annual precipitation, and temperature seasonality had the highest average contribution over the ten replications and four models, indicating that the suitable habitat for the species in northern Iran is characterized by both landscape-based (i.e. woody alpine habitats) and climatic variables.

We assessed the amount of protection granted to *G*. *h*. *caucasicus* in northern Iran by overlaying the Biomod suitability map with areas occupied by the lineages and the newest map of protected areas network of Iran, including national parks, protected areas, and wildlife refuges (Fig. 5). We estimated the effectiveness of the current protected area network to be 29.7% for KD-EA, 39.1% for LarNP-CA, 57.6% for CA, and 24.8% for WA-Az.

## Discussion

### Reassessment of the taxonomic status of the Caucasian pit viper (*G*. *h*. *caucasicus*)

Recently, a number of taxa formerly viewed as subspecies of *G*. *halys* have been raised to full species rank based on genetic evidence. Among these are *G*. *h*. *caraganus*, submitted for reconsideration as a species (*G*. *caraganus*) (Eichwald, 1831) by Wagner *et al*.^17^ and two other subspecies (*G*. *h*. *cognatus* and *G*. *h*. *stejnegeri*), elevated to full species rank by Shi *et al*.^18,19^. Simonov *et al*.^40^ proposed the elevation of all currently recognized subspecies, *G*. *halys* to species rank (including *G*. *h*. *caucasicus*), while emphasizing that “*G*. *saxatilis”* and *G*. *intermedius* are synonymous^20^, and that *G*. *lijianlii* could be synonymised with *G*. *changdaoensis* due to the absence of genetic differentiation in their mitochondrial genes.

Genetic distances among taxa of the *G*. *halys/G*. *intermedius* complex (clade F in Shi *et al*.^18^) indicate that the distances vary from 1.0–5.4% (Fig. S1 and the blue cells in Table S3), which conforms to the results of Shi *et al*.^19^. They also estimated the genetic distance among *G*. *stejnegeri*, *G*. *cognatus*, and *G*. *h*. *halys* to range from 2.7–5.0%^19^. However, the average genetic distance between the four Iranian clades of *G*. *h*. *caucasicus* and the other species varies from 3.7–5.6% (Fig. S1 and the grey cells in Table S3), which is even greater than the distance observed by Shi *et al*.^19^ between *G*. *cognatus* and *G*. *stejnegeri* (2.7–4.84%). In addition, the genetic distance between *G*. *changdaoensis*, *G*. *intermedius*, *G*. *shedaoensis*, *G*. *cognatus*, *G*. *stejnegeri*, and *G*. *caraganus* varies from 1.0%–5.4% (the blue cells in Table S3), while the average genetic distance among the four Iranian clades of *G*. *h*. *caucasicus* and the other taxa in the complex varies from 4.2–5.2% (the grey cells in Table S3). This is also noticeable in the haplotype network (Fig. 2). The results suggest that *G*. *h*. *caucasicus* could be considered as a distinct species. This is reconfirmed by our Bayesian multi-locus species delimitation approach (under different scenarios), which strongly supported each of the nine previously delimited taxa in the complex with maximum Bayesian posterior probability (P = 1).

However, the pairwise genetic distance among the Iranian lineages of *G*. *h*. *caucasicus* (clades KD-EA, WE-Az, LarNP-CA, and CA) drops to 1.5–2.9%, (Fig. S1 and the green cells in Table S3), as Bayesian posterior probabilities of our second scenario dropped to 0.93%–95% under all different priors.

Furthermore, Khani *et al*.^29^ separated three populations of *G*. *caucasicus* in the Alborz Mountains based on seven metric and 21 meristic traits. Their study showed that populations of eastern Alborz, Lar National Park and western Alborz, corresponding respectively to KD-EA, LarNP-CA, and WA-Az in our study, are significantly different with respect to morphological traits. This means that they failed to differentiate between LarNP-CA and CA, apparently due to the lack of samples from Central Alborz (including western Mazandaran and eastern Gilan provinces).

However, the results of a study conducted by Malek-Mohammadi *et al*.^25^ based on a 775 bp D-Loop dataset did not find any significant distinction among populations of *G*. *caucasicus* in the Alborz Mountains^25^. Another study on the phylogenetic relationships of *G*. *caucasicus* in Iran using 629 bp of the cyt *b* gene from 16 individuals in north-east of Iran (Khorasan province) and Central Alborz (Lar National Park and Gachsar area) concludes that the species belongs to the *G*. *halys/G*. *intermedius* complex, within which controversial phylogenetic relationships still remain^26^.

They also suggest that all samples of *G*. *caucasicus* in northern Iran belong to a single population. However, the distinction between the *G*. *caucasicus* clade and other closely related clades (*G*. *intermedius*, *“G*. *saxatilis”*, and *G*. *shedaoensis*) have low support (Pp = 0.36 and bootstrap values = 59%^26^). The genetic distance between *G*. *caucasicus* and the species previously mentioned was much lower in comparison to our findings (0.6%–0.9% versus 4.2%–5.2%). This discrepancy may result from taxonomic misidentification for the sequences that Rastegar-Pouyani *et al*.^26^ obtained from GenBank. As a result of the high taxonomic uncertainty within this group, along with difficulties with morphological identification of the taxa, many GenBank entries for this complex have erroneous taxonomic names. This mislabeling of species names may result in incorrect estimation of genetic distances between taxa.

Thus, in the light of molecular and morphological evidences, we argue that *G*. *h*. *caucasicus* in northern Iran should be elevated to species rank and further referred to as *G*. *caucasicus* (Nikolsky, 1916). We therefore refer to *G*. *caucasicus* as such and use this labeling throughout subsequent text.

### Allopatric divergence and dispersal during the Pleistocene oscillations

#### Diversification of the *Gloydius* genus

In view of our biogeographical results, the DIVALIKE + J model was regarded as the most probable pattern of dispersal, vicariance, and extinction for the *Gloydius* genus in the Palaearctic (Table S2, Scenario_HB_ 2). We found no evidence of ancestral range switching throughout the distribution range of the complex. Accordingly, this model appeared as the best biogeographical model of ancestral expansion for the complex^41–43^. During the Pleistocene, historical expansion of *Gloydius* populations was in alignment with dispersal patterns of other species occupying similar geographical distribution ranges. They all dispersed in a similar manner from the eastern Palearctic toward central regions and lower latitudes, a movement which led to the establishment of new populations in a discrete refugium^44,45^. Moreover, the absence or minor levels of gene flow among eastern (*G*. *h*. *halys*, *G*. *stejnegeri*, and *G*. *cognatus*) and central (*G*. *caucasicus*, *G*. *rickmersi*, and *G*. *caraganus*) Palearctic species around 1.0–2.8 Mya was proposed as the best biogeographical scenario based on AIC and AICc weight (Table S2), which could be indicative of vicariance due to geographical isolation of populations in multiple and isolated refugia.

During the Last Glacial Maximum (LGM), glaciers advanced upon lands toward lower latitudes and covered more than 30% of the earth’s surface, creating a major impact on the overall climate of the planet, including Iran’s^46^. Some geomorphological evidence suggests that Iran has substantially and prolongedly altered during the Quaternary period^47^. According to Ehlers^48^, the climate of Iran has experienced a severe reduction in temperature, and consequently, a moderate increase in precipitation in montane habitats during the early Würm (approximately 100 kya). Kehl^49^ notes that the Quaternary climate in northern and western Iran remained dry and cold during glacial periods, while it was warm and wet during interglacial periods. The effects of glacial periods on mountains of Iran (including Azerbaijan, Kurdistan, Alam-Kouh, and Damavand) and adjacent glacial mountains (Shirkouh, Zardkouh, Kerman mountains, Southern Alborz, and Northern Khorasan), and the presence of Loess soils in central Iran provide evidence for a past cold climate^49–52^. Major glacial centers were located in Alam-kouh^53^, Sabalan^54^, borders of Iran-Turkey and Iran-Iraq^55,56^, Zardukh^57^, and possibly the Dinar Mountain in central Zagros^49^. It has been estimated that during the LGM, temperature fluctuations in the Alborz and Zagros mountains, as well as central Iran ranged from five to eight degrees Celsius^49^ colder than present.

#### Diversification of *Gloydius caucasicus*

The BEAST analyses revealed that the divergence of *G*. *caucasicus* from other species of the complex occurred in the lower Pleistocene (1.89 Myr, 95% HPD: 1.20–2.70 Myr; Fig. 3), while the divergence among populations of *G*. *caucasicus* in northern Iran comprised three phases of divergence during the Pleistocene, which highlights the effects of climate oscillations on isolation of populations. During the early Pleistocene (2.5–1.84 Myr), Earth’s climate was cooler and drier compared to the mid-Pliocene^58^. As a result of the reduction in Earth’s temperature, suitable habitats were confined to more southerly latitudes (40–50 degrees of latitude) and lower elevations^59^. Given that *G*. *rickmersi* and *G*. *caraganus* are sister clades to *G*. *caucasicus* (Fig. 1), it could be inferred that as a consequence of the cooling of high latitudes, one could expect gene flow to be driven toward more southerly latitudes such as Afghanistan and northeastern Iran (Fig. 4).

The first divergence due to vicariance in populations of *G*. *caucasicus* in northern Iran is estimated to have occurred around 1.25 Myr (95% HPD: 0.73–1.83 Myr). At that time, populations of central and western Alborz diverged from populations of the north-east. Results of ABC analyses suggested a bi-directional gene flow from northeastern Iran to northwestern and southern Azerbaijan through northern and southern slopes of the Alborz, strikingly similar to the intraspecific divergence of *Montivipera raddei* species occurring 1.88 Myr in the Alborz, north-western and Zagros Mountains^13^. Meanwhile, the second divergence occurred between clades of central Alborz and western Alborz (1.09; 95% HPD: 0.64–1.64 Myr). Most likely, following the first glacial maximum in the lower Pleistocene (1.84 Myr), a warm interglacial period contributed to the expansion of populations toward central Alborz via the northern slopes, and toward western Alborz and mountains of southern Azerbaijan via the southern slopes of the Alborz. This could be regarded as the existence of multiple potential refugia for *G*. *caucasicus* populations along the Alborz, conforming to the lineages identified in these areas. The last divergence within the species occurred between populations of central Alborz approximately 0.68 Myr (95% HPD: 0.34–0.89 Myr), prior to the Günz glacial period (0.62–0.67 Myr).

It could be concluded that populations of *G*. *caucasicus* in northern Iran have undergone multiple expansions and contractions during glacial and interglacial periods. Nevertheless, no shared haplotypes were observed between the lineages. This can be explained upon the assumption that the gene flow from northeastern to central and western Alborz was unidirectional, meaning that no genes were transferred back to ancestral populations. It could be presumed that the warming of the earth from the early Holocene to the present might have contributed to population fragmentation in mountains and led to a breakdown of gene flow between the different regions.

Finally, our results could underline the existence of multiple glacial refugia in the Alborz during climatic oscillations of Pleistocene. This complies with the results of previous studies^60–66^, which suggested the Hyrcanian forests as an isolated refugium during Quaternary oscillations. Isolation of organisms eventually gave rise to vicariance in a middle-sized geographical area in the central Palearctic. Such multiple glacial refugia contributed to the interruption or reduction of gene flow, and consequently increased genetic drift, resulting in formation of endemic haplotypes and new subspecies^67^, which, following the last glacial period when the climate became more favorable, expanded toward suitable habitats and shaped the current patterns of distribution.

### Conservation Units and Management Propositions

Conservationists have long been in a quandary regarding the continuing controversy over the definition of conservation units at the species or subspecies level, stressing the need for its reconsideration^39,68,69^. Evolutionary significant units (ESUs) generally refer to taxa that merit independent conservation management because they have evolved separately^70,71^.

Among the many definitions assigned to the concept of ESUs, we chose the one adopted by Fraser and Bernatchez^39^, as its overall focus remains on isolated populations. They state that lineages with particularly restricted patterns and levels of intraspecific gene flow are to be considered as ESUs for conservation. Therefore, we proposed the four isolated lineages of *G*. *caucasicus* identified in northern Iran as four ESUs including (i) KD-EA ESU comprising the Hezar Masjid Mountains, Khorasan, and Golestan provinces, (ii) LarNP-CA ESU including Lar area as well as Mazandaran province, (iii) CA ESU including northern and western slopes of the Alborz Mountains in western Mazandaran and eastern Gilan provinces, and (iv) WA-Az ESU comprising western Gilan and Ardebil provinces.

The International Union for Conservation of Nature (IUCN) has classified *G*. *monticola* (Likiang Pit Viper) from China as Data Deficient (DD), “*G*. *saxatilis”* (Rock Mamushi) from China, Korea, and Russia as Least Concern (LC), and *G*. *shedaoensis* (Shedao Island Pit Viper) from China as Vulnerable (VU). However, the conservation statuses of the other species in the two complex groups of *G*. *blomhoffi* and *G*. *halys/G*. *intermedius* (including *G*. *caucasicus*) have received little consideration. Although our results confirmed that about 37.8% of suitable *G*. *caucasicus* habitats are located within the network of protected areas in northern Iran, this species is currently threatened by various factors such as agricultural development, overgrazing of livestock, destruction of rangelands, hunting and killing by local people and/or tourists, mortality due to vehicle collisions on roads, restricted movement of individuals among population patches, and large-scale hunting and capturing for vaccine and serum production^26^ (about 1000 capturing licenses are certified annually). Unfortunately, hunting/capturing of this species is mostly done when snakes emerge from hibernation and have not yet had the chance to reproduce. In general, populations of this species have experienced dramatic declines over the past decades, to the extent that snake catchers complain that it is now hardly possible to locate well-populated sites for this species.

However, *G*. *caucasicus* is not listed as a protected species according to the Department of Environment of Iran, accompanying many other reptiles that have been largely neglected from the list of protected species. Moreover, the lack of awareness and limited knowledge regarding venomous snakes has instilled deep fear in local people and even park rangers to such an extent that they would habitually kill a snake upon their very first encounter. Therefore, we recommend that the Department of Environment of Iran should (i) establish and declare new protected areas throughout the distribution range of the Caucasian pit viper, according to the four lineages identified in this study, (ii) establish safe zones in current protected areas that cover suitable habitats of the Caucasian pit viper; (iii) prevent or reduce legal hunting and venom collection from populations of the Caucasian pit viper until populations have recovered, (iv) list the species as protected under laws of the Department of Environment of Iran, and reduce its international illegal trade by the addition of the species to the CITES appendices.

## Material and Methods

### Sampling, PCR amplification, and DNA sequencing

**Figure 6:**
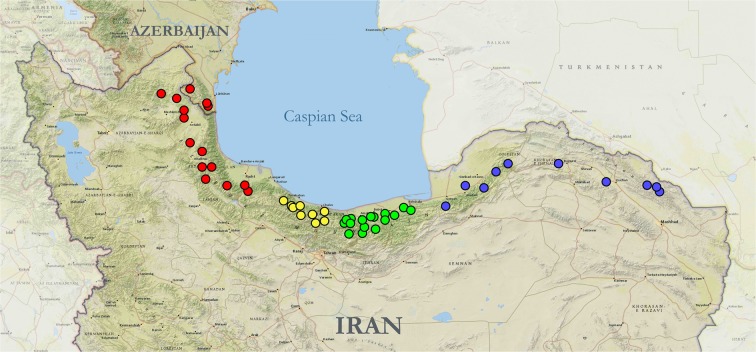
Localities for 53 samples used in the study from Iran and Azerbaijan. Colours correspond to phylogenetic lineages of *G*. *h*. *caucasicus* along the Alborz Mountains, namely Kopet Dagh-Eastern Alborz (blue, KD-EA), Lar National Park-Central Alborz (green, LarNP-CA), Central Alborz (yellow, AC), and Western Alborz-Azerbaijan (red, WA-Az). Map source: ESRI. The map was generated using ArcGIS 10.2 by ESRI (available online at: http://www.esri.com/).

We obtained sequence information for 41 individuals of the Caucasian pit viper, representing 15 regions from northeastern to northwestern Iran and Azerbaijan (Fig. 6, Table S4). Tissue samples contained three clips from the outer edge of ventral scales for each specimen, except two museum samples from Azerbaijan, for which muscle tissue was used. Captured snakes were released into their capture location immediately. All methods were performed in accordance with the relevant guidelines and regulations. This study was licensed by the Iranian Department of Environment under permits No. 94/6049 and 96/3631.

Total genomic DNA was extracted from tissue samples using a Qiagen DNeasy Tissue kit (Qiagen, Courtaboeuf, France) or by phenol/chloroform protocol^72^. We amplified two fragments of the mitochondrial genome including 1125 base pairs (bp) of the cyt *b* and 678 bp of the ND4. Furthermore, we partially sequenced one nuclear proto oncogene c-mos (567 bp), which evolves at a slower rate than mtDNA^73^. All genes were amplified by polymerase chain reaction (see Table S5 for PCR protocols) and sequenced using primers designed by previous studies (L14910/H16064^74^ and ND4/Leu^75^ for the two mtDNA genes, and S77 and S78 for c-mos^76^). PCR products were sent to Eurofins Genomics (Ebersberg, Germany) or SYNTOL Company (Moscow, Russia) for sequencing on an ABI 3730 automated DNA sequencer (Applied Biosystems).

### Sequence alignment and data analyses

Sequences of samples were examined using SeqScape version 2.6 (Applied Biosystems). We also obtained 56 sequences (comprising 13 species) from GenBank^17–19,26,27,77–79^ (Table S4). All sequences were aligned using ClustalW implemented in MEGA v.6^80^. Protein coding sequences were converted into amino acid residues to check for stop codons as a result of pseudogene generation. We used DnaSP version 5.0^81^ to calculate mitochondrial diversity indices including haplotype and nucleotide diversities, number of haplotypes, and polymorphic sites. Nucleotide composition and genetic distances were analyzed using the uncorrected genetic distance including 1000 bootstraps in MEGA v.6.

### Phylogenetic relationship and Taxonomic assessment

#### Species tree estimation

We concatenated the datasets of mtDNA (cyt *b* and ND4) + nDNA (c-mos) including 2370 bp for 53 samples of *G*. *h*. *caucasicus*, nine samples of *G*. *h*. *halys*, seven samples of *G*. *cognatus*, five samples of *G*. *stejnegeri*, two samples of *G*. *intermedius*, three samples of *G*. *caraganus*, two samples of *G*. *rickmersi*, six samples of *G*. *shedaoensis*, and two samples of *G*. *changdaoensis*. We used PartitionFinder 1.1.1^49^ to identify the best partitioning schemes and model of sequence evolution for each partition using the “greedy” algorithm and the Bayesian Information Criterion (BIC). Bayesian Inference (BI) and Maximum Likelihood (ML) phylogenetic analyses were carried out using the selected scheme. For the BI analysis, we used MrBayes 3.1.2^82^ and the analysis was run using one cold and three heated chains (MC3) for 40 million generations, sampling every 1000th generation and discarding the first 25% of the trees as burn-in. Convergence was examined using Tracer v1.5^83^ and checked with the convergence diagnostic parameters performed in MrBayes. A ML phylogenetic analysis was carried out using the selected model in IQ-TREE version 1.6.2^84,85^ and 1000 non-parametric bootstrap replicates.

#### Haplotype network

The concatenated sequences of the two mitochondrial genes in this study were 1803 bp in length, whereas the majority of sequences downloaded from GenBank were shorter. To avoid any potential bias caused by uneven sequence lengths among samples, we used an evenly-matched length of sequences for our analyses. We made an mtDNA matrix with 70 individuals (39 samples of *G*. *caucasicus* and 31 samples of the other taxa of *G*. *halys/G*. *intermedius* complex) with 1618 bp. We then developed a haplotype network in order to visualize the genetic relationships among the complex haplotypes. Haplotype networks were estimated with TCS^86^ implemented in PopART (http://popart.otago.ac.nz).

#### Spatial analyses of genetic variability

We analyzed population structure and determined the amount of mixture between population clusters on our concatenated mtDNA + nDNA dataset (including 2370 bp) using the Bayesian Analysis of Population Structure software (BAPS v.6.0)^87,88^. We allowed K (number of clusters) to vary from 1–9 in order to calculate the best value for K. Then, an AMOVA was executed through Arlequin version 3.1^89^ with populations grouped into the best number of K identified for population clusters. Also, we used F-statistics with 10000 permutations to estimate the proportion of genetic variability among different fixation indices, FST (i.e. variance among populations relative to the total variance).

#### Barcoding gap analysis

We used the concept of the ‘barcoding gap’ to determine the threshold of species level in the *G*. *halys/G*. *intermedius* complex using the mtDNA dataset. We calculated genetic distances among the taxa of the complex (clade F in Shi *et al*.^18^) along with the four lineages for *G*. *caucasicus* in northern Iran, using an evenly-matched length of sequences from our mtDNA dataset. Pairwise inter- and intraspecific genetic distances were calculated using uncorrected p-distances by MEGA v.6^80^. Then, we plotted frequency distribution histograms of pairwise inter- and intraspecific distances.

#### Bayesian species delimitation

We adopted a Bayesian multi-locus species delimitation approach implemented in BPP 3.1^90–92^ to verify the speciation patterns within the complex based on our concatenated mtDNA + nDNA dataset (gene-partitioned), with the ML and BI topologies from this study serving as the guide tree. We tested two different scenarios: (i) considering the nine major clades corresponding to the nine taxa of the complex obtained by the BI and ML analyses, and (2) including 12 putative clades (the four Iranian clades of *G*. *caucasicus* and the remaining species of the complex).

This method estimates population size (θ) and divergence time (τ) parameters and then applies a reverse-jump MCMC (rjMCMC) algorithm to calculate posterior probabilities for species delimitation. BPP assumes that there is neither recombination within a locus nor gene flow between species^92^. It also assumes neutral clock-like evolution and employs the JC69 mutation model; therefore, it could only be used for closely related species with sequence divergences not much higher than 10%^93^. We evaluated the neutrality of the two mtDNA genes by Hudson-Kreitman-Aguadé tests (HKA)^94^ in DnaSP v 5.10^81^. Moreover, we assessed our nDNA recombination through the pairwise homoplasy index (PHI) test^95^ in Splitstree4^96^.

A Dirichlet distribution was employed with α = 2 to compensate for variation in mutation rates among loci. A gamma prior (G) was applied to specify the population size parameter $({\rm{\theta }})$ and root age (τ0) of the species tree. As BPP is sensitive to the prior values^97^, we made three replicate runs under three different combinations of gamma-distributed priors for ancestral θ and root age (τ0)^98–101^: (i) assuming relatively large ancestral population sizes and deep divergences, ${\rm{\theta }}$ ~ G(1,10) and τ0 ~ G(1,10); (ii) assuming relatively small ancestral population sizes and shallow divergences among species, ${\rm{\theta }}$ ~ G(2,2000) and τ0 ~ G(2,2000); and (iii) a conservative combination of priors that could fit models with fewer species ${\rm{\theta }}$ ~ G(1,10) and τ0 ~ G(2,2000). Each analysis of 10^6^ rjMCMC generations was run twice from different starting seeds (+1 and −1) with a burn-in period of 105 using algorithm 1 (α = 2 and m = 1). For all speciation events, we conservatively regarded speciation probability values > 0.95 as strong support.

### Historical biogeography analysis

#### Molecular dating and divergence time

We estimated divergence times using BEAST 1.8.0^102^ on our concatenated mtDNA + nDNA dataset with three calibration points including (i) divergence of three populations of the genus *Porthidium* in South America, some 3.5 Mya^103^, using a normal distribution model (mean = 3.5 Mya, SD = 0.51 Myr, and 95% CI = 2.5–4.5 Myr), (ii) divergence between *Crotalus* and *Sistrurus* before 9 Mya^104^, using a lognormal prior model with a zero offset of 9 Mya (mean = 1 Mya and SD = 1 Myr)^105^, and finally (iii) divergence of the Eurasian vipers clade (genera *Macrovipera*, *Montivipera*, and *Vipera*) about 20 Mya suggested by fossil data^106,107^, using a lognormal prior model with a zero offset of 17 Myr (mean = 1 Mya, SD = 1 Myr, and 95% CI = 17–36 Myr)^105^.

We also included some sequences of four species of *Montivipera*, two species of *Macrovipera*, two species of *Vipera*, two species of *Sistrurus*, four species of *Crotalus*, four species of *Porthidium*, as well as seven outgroups (*G*. *brevicaudus*, *G*. *ussuriensis*, *G*. *blomhoffi*, *G*. *strauchi*, *G*. *rubromaculatus*, *G*. *liupanensis*, and *G*. *tsushimaensis*) in our dataset as calibration points (see Table S4). PartitionFinder 1.1.1^49^ was used to select the best data partition and evolutionary models in our molecular dating. We also adopted the Birth-Death Process model because it is a proper model when sequences from different species are included in a dataset^108^. The fitness of three molecular clock models (Strict, Exponential relaxed, and Lognormal relaxed) was tested by Bayes factor analysis in Tracer v1.5^83^, using value of 2LnBF^109^. Each analysis was performed using two independent runs of 40 million generations, sampled every 1000 generations, with the first 25% discarded as burn-in. Tracer was used to evaluate acceptable levels of MCMC chain mixing, the stationary likelihoods and appropriate lengths of burn-in (25%), as well as to estimate effective sample sizes for all parameters.

#### Historical biogeography reconstruction

Using the concatenated mtDNA + nDNA dataset, we inferred the ancestral range and colonization history of the complex through the Palaearctic using the R package BioGeoBEARS^110^ under three models of biogeographical range expansion (i) the Dispersal-Extinction-Cladogenesis (DEC), (ii) Dispersal-Vicariance (DIVALIKE), and (iii) Bayesian inference (BAYAREALIKE). We also applied the jump dispersal parameter J to these models, and likelihood values of all six models (DEC, DEC + J, DIVA-like, DIVA-like + J, BAYAREA-like, BAYAREA-like + J) were compared with the Akaike Information Criteria (AICc). We defined seven regions based on knowledge of species distribution, namely A: *G*. *changdaoensis* and *G*. *intermedius*, B: *G*. *caraganus*, C: *G*. *rickmersi*, D: *G*. *h*. *caucasicus*, E: *G*. *h*. *halys*, F: *G*. *shedaoensis*, and G: *G*. *cognatus* and *G*. *stejnegeri* (Fig. 4). We used BEAST 1.8.2^58^ to construct an ultrametric tree, then pruned all outgroups using Mesquite v.3.04^111^. We chose four time slices (0–0.30, 0.30–1.20, 1.20–2.50 and 2.50–3.45 Mya), corresponding to divergence times within the complex. First, we ran the S0 scenario in which dispersal between regions was not penalized. Then, we tested four alternative scenarios (Scenario_HB_ 0–3), where we tested colonization of the complex lineages from other adjacent regions (Fig. 4). We also estimated the geographic location of the ancestors of the *G*. *halys/G*. *intermedius* complex, employing a statistical method implemented in PhyloMapper 1b1^112^, optimized by 10,000 replications.

#### Evolutionary hypothesis testing

**Figure 7:**
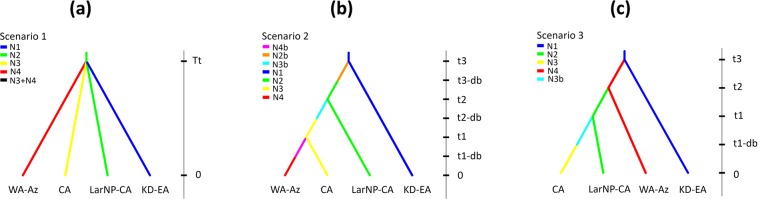
Population trees obtained using coalescent simulations, representing the three biogeographic hypotheses of diversification within *G*. *h*. *caucasicus*. (**a**) The null model of the fragmentation of a widespread ancestor or a single-refugium population, and (**b**,**c**) the alternative multiple-refugial/vicariance models, where b and c present one way and two-way gene flows, respectively.

We applied coalescent simulations to test three alternative hypotheses (Scenarios_EH_ 1–3) regarding the demographic history of *G*. *h*. *caucasicus*, using an Approximate Bayesian Computation (ABC) approach on our concatenated mtDNA + nDNA dataset. In the first scenario tested, (i) we considered fragmentation of a single ancestral source population, which supposes that the four lineages could have diverged from a single ancestral refugium, approximately, at most, up to the Last Glacial Maximum, consistent with Weichselian (in Scandinavia and northern Europe), Würm (in Alps), and Wisconsin (in North America) glaciations, and then colonized different climatic and environmental niches throughout the Alborz Mountains (Fig. 7a). In the two other scenarios, we supposed multiple glacial refugia (or long-term geographical isolation among regions), which predicts that the four lineages may have developed under the effects of diversification from multiple refugia in northern Iran and two demographic history scenarios were tested to demonstrate whether (ii) the gene flow from northeastern Iran to western Alborz and Azerbaijan has experienced a one-way flow through the northern slopes, or (iii) a two-way gene flow through the northern and southern slopes of the Alborz Mountains. In a one-way gene flow, we considered an incursion from KD-EA to LarNP-CA, then to CA, and proceeding to WA-Az (Fig. 7b), whereas in a two-way gene flow, we tested a diversification from KD-WA to LarNP-CA, then to CA through the northern slopes of the Alborz Mountains, along with another diversification from KD-EA to WA-Az through the southern slopes of the Alborz Mountains (Fig. 7c).

We used an Approximate Bayesian Computation (ABC) approach in the program DIYABC 2.1.0^113^ to obtain the relative probabilities for the competing hypotheses. In this approach, summary statistics of our molecular data were calculated and compared to the dataset simulated earlier based on the modelled scenarios. Then, Euclidean distances between our simulated dataset and the observed dataset were calculated by a local linear regression. Finally, we only kept 10 subsets of the closest 2% of our simulated data to the observed data in order to compute posterior distributions, which enabled us to prioritize our modelled scenarios based on approximate marginal likelihoods and find the best-fit model. We used summary statistics including number of haplotypes as well as segregating sites, mean pairwise differences, mean between-sample pairwise differences, number of private segregating sites, and FST values.

We also used uniform priors with a lower and an upper bound for population size of 10 to 7 × 10^5^, and divergence times of 2.46 × 10^5^ for t1, 4.30 × 10^5^ for t2, 4.86 × 10^5^ for t3, and 6.86 × 10^5^ for Tt generations in the past. Furthermore, we assumed a generation time of 3 years, the age of sexual maturity for *G*. *brevicaudus*^6^, as a closely related species to *Gloydius*^114^. In addition, Yakovleva^115^ has reported the minimum body length of the sexually matured specimens of *G*. *halys* sensu lato in Kyrgyzstan as 330 mm, which corresponded to the age of 3 years in our data. Moreover, all populations could include a discrete size-change event. We estimated the posterior probability of each hypothesis using both the direct approach (on the 500 closest datasets) as well as the logistic approach (on the 1% closest to the observed data). Then, to calculate posterior distributions of parameters, we used a local linear regression on 1% of the accepted closest simulated data merely based on the most likely hypothesis. To evaluate the strength and accuracy of our ABC model selection, we simulated 1000 test datasets (pseudo-observed datasets) under each of the competing hypotheses and calculated the probability of type I and type II errors, assuming the defined priors in the historical model.

We determined the effective population sizes (*Ne*) using $\theta$-values estimated by the ML and the coalescent-theory approach in MIGRATE 3.2.1^116^. We ran the analysis with 10 short chains of 200,000 steps, followed by three long chains of two million steps, sampling every 20 steps following a burn-in of 10,000 steps. Then, we calculated *Ne* using the equation for maternally inherited mtDNA ($\theta$ = *Ne* $\mu$). We considered $\mu =3.9\times {10}^{-8}$, based on the mean rate of sequence evolution of approximately 0.01306 substitutions per Myr, using BEAST 1.8.2^102^.

### Species Distribution Modelling

We first compiled data of the species occurrence (84 points) with 10 climatic, land cover, and physiographic variables to build a species distribution model (SDM) for the species. For climatic variables, we used the WorldClim dataset^117^, a set of 19 climatic variables with ~1 km resolution. Due to the high correlation between the climatic variables, we first calculated pairwise correlation coefficients among the variables and then screened them to low correlated (*r* < 0.75) variables. Accordingly, we obtained Bio1 (annual mean temperature), Bio4 (temperature seasonality), Bio5 (maximum temperature for the warmest month), Bio12 (annual precipitation) and Bio13 (maximum precipitation for the wettest month). Land cover variables including distance to forest patches, distance to herbaceous cover with shrubs and sparse trees, and distance to herbaceous cover, were generated in ArcMap 10.3 based on cover types of Globcover v. 2.1^118^. Based on the Shuttle Radar Topography Mission (SRTM) elevation model (http://srtm.csi.cgiar.org), we also used altitude and topographic roughness (i.e., standard deviation of altitude for all raster cells within a 5 × 5 km moving window) as the most important variables depicting physiographic heterogeneity.

To generate a habitat suitability map, we then conducted four SDM algorithms, including generalized linear models (GLM), generalized boosting models (GBM), maximum entropy (MaxEnt), and random forest (RF), and combined them into a final ensemble model using *Biomod 2* package in R v.3.3.2^119^. To reduce bias caused by randomly selected occurrence points for model construction, we replicated the modelling based on a 10-fold cross-validation approach, using a different subset of 25% of the occurrence records to test each model. Model performance was evaluated based on the area under the curve (AUC) of a receiver operating characteristic (ROC) plot and the true skill statistic (TSS). The final ensemble model was obtained by weighted averaging the individual models proportional to their AUC scores. Finally, we used the boundaries of populations, estimated from BAPS, to separate the boundary of the continuous suitability map of the lineages from northeastern to northwestern Iran.

## Supplementary information

Supplementary information

## References

[1] Hewitt GM (1999). Post-glacial re-colonization of European biota. Biol. J. Linn. Soc..

[2] Hewitt GM (1996). Some genetic consequences of ice ages, and their role in divergence and speciation. Biol. J. Linn. Soc..

[3] Avise, J. C. Phylogeography: The History and Formation of Species. (Harvard University Press, 2000).

[4] Cox, C. B., Moore, P. D. & Ladle, R. Biogeography: an ecological and evolutionary approach. (John Wiley & Sons, 2016).

[5] Ursenbacher S (2006). Phylogeography of the asp viper (*Vipera aspis*) inferred from mitochondrial DNA sequence data: evidence for multiple Mediterranean refugial areas. Mol. Phylogenet. Evol..

[6] Ding L, Gan XN, He SP, Zhao EM (2011). A phylogeographic, demographic and historical analysis of the short‐tailed pit viper (*Gloydius brevicaudus*): evidence for early divergence and late expansion during the Pleistocene. Mol. Ecol..

[7] Runck AM, Cook JA (2005). Postglacial expansion of the southern red‐backed vole (*Clethrionomys gapperi*) in North America. Mol. Ecol..

[8] Sagheb Talebi, K., Sajedi, T. & Pourhashemi, M. Forests of Iran: A Treasure from the Past, a Hope for the Future. (Springer, 2013).

[9] Alai-Taleghani, M. Geomorphology of Iran. (Ghomes Publications, 2015).

[10] Guest B, Horton BK, Axen GJ, Hassanzadeh J, McIntosh WC (2007). Middle to late Cenozoic basin evolution in the western Alborz Mountains: Implications for the onset of collisional deformation in northern Iran. Tectonics..

[11] Khosrow-Tehrani, K. Stratigraphy and geological events. (University of Tehran Press, 2013).

[12] Rajaei SH (2013). Quaternary refugia in southwestern Iran: insights from two sympatric moth species (Insecta, Lepidoptera). Org. Divers. Evol..

[13] Behrooz R (2018). Conservation Below the Species Level: Suitable Evolutionarily Significant Units among Mountain Vipers (the *Montivipera raddei* complex) in Iran. J. Hered..

[14] Yousefi M (2015). Upward altitudinal shifts in habitat suitability of mountain vipers since the last glacial maximum. PLOS ONE..

[15] Dynesius M, Jansson R (2000). Evolutionary consequences of changes in species’ geographical distributions driven by Milankovitch climate oscillations. P. Natl. A. Sci..

[16] Orlov NL, Sundukov YN, Kropachev II (2014). Distribution of pitvipers of *Gloydius blomhoffii* complex in Russia with the first records of *Gloydius blomhoffii blomhoffii* at Kunashir Island (Kuril archipelago, Russian Far East). Russ. J. Herpetol..

[17] Wagner P, Tiutenko A, Mazepa G, Borkin LJ, Simonov E (2016). Alai! Alai!–a new species of the *Gloydius halys* (Pallas, 1776) complex (Viperidae, Crotalinae), including a brief review of the complex. Amphibia-Reptilia..

[18] Shi J (2017). A new moth-preying alpine pit viper species from Qinghai-Tibetan Plateau (Viperidae, Crotalinae). Amphibia-Reptilia..

[19] Shi J, Yang D, Zhang W, Ding L (2016). Distribution and infraspecies taxonomy of *Gloydius halys-Gloydius intermedius* complex in China (Serpentes: Crotalinae). Chin. J. Zool..

[20] Orlov NL, Barabanov AV (1999). Analysis of nomenclature, classification, and distribution of the *Agkistrodon halys*–*Agkistrodon intermedius* complexes: a critical review. Russ. J. Herpetol..

[21] David P, Ineich I (1999). Les serpents venimeux du monde: systématique et répartition. Dumerilia..

[22] Gumprecht, A. Die Weisslippen-Bambusotter, *Cryptelytrops albolabris* (*Trimeresurus albolabris*). (Art für Art, Natur und Tier Verlag, 2004).

[23] Orlov NL, Barabanov AV (2000). About type localities for some species of the genus *Gloydius* Hoge et Romano-Hoge, 1981 (Crotalinae: Viperidae: Serpentes). Russ. J. Herpetol..

[24] Simonov E, Wink M (2012). Population genetics of the Halys pit viper (*Gloydius halys*) at the northern distribution limit in Siberia. Amphibia-Reptilia..

[25] Malek-Mohammadi A, Rastegar-Pouyani E, Todehdehghan F (2017). Genetic relationship between different populations of the *Gloydius halys caucasicus* (Nikolsky 1916) of mountainous areas of the Iran. SCIREA J. Agr..

[26] Rastegar-Pouyani E, Oraie H, Khosravani A, Akbari A (2018). Phylogenetic position of Iranian pitvipers (Viperidae, Crotalinae, *Gloydius*) inferred from mitochondrial cytochrome *b* sequences. Trop. Zool..

[27] Xu Y (2012). Molecular phylogeny of the genus *Gloydius* (Serpentes: Crotalinae). Asian Herpetol. Res..

[28] David, P. & Vogel, G. An updated list of Asian pitvipers and a selection of recent publications (ed. Visser, D) In: Asian Pitvipers, Breeding Experience and Wildlife. (ECO Wear & Publishing, 2015).

[29] Khani S, Kami HG, Rajabizadeh M (2017). Geographic variation of *Gloydius halys caucasicus* (Serpentes: Viperidae) in Iran. Zool. Middle East..

[30] Simonov E (2009). Differences in habitat use, daily activity patterns and preferred ambient temperatures of adult and neonate *Gloydius halys halys* from an isolated population in southwest Siberia: preliminary data. Herpetol. Notes..

[31] Mozafari SZ, Shiravi A, Todehdehghan F (2012). Evaluation of reproductive parameters of vas deferens sperms in Caucasian snake (*Gloydius halys caucasicus*). Vet. Res. For..

[32] Shakoori S, Todehdehghan F, Shiravi A, Hojati V (2015). The assessment of captive breeding in the Caucasian viper (*Gloydius halys caucasicus*) in Iran. J. Entomol. Zool. Stud..

[33] Hoser R (2013). A review and rearrangement of pitviper genera (Serpentes: Viperidae: Crotalinae). Australas. J. Herpetol..

[34] Kaiser H (2013). Best practices: in the 21st century, taxonomic decisions in herpetology are acceptable only when supported by a body of evidence and published via peer-review. Herpetol. Rev..

[35] Palandačić A, Naseka A, Ramler D, Ahnelt H (2017). Contrasting morphology with molecular data: an approach to revision of species complexes based on the example of European *Phoxinus* (Cyprinidae). BMC Evol. Biol..

[36] Deans AR, Yoder MJ, Balhoff JP (2012). Time to change how we describe biodiversity. Trends Ecol. Evol..

[37] Casacci LP, Barbero F, Balletto E (2014). The “Evolutionarily Significant Unit” concept and its applicability in biological conservation. Ital. J. Zool..

[38] Mace GM (2004). The role of taxonomy in species conservation. Philos. T. Roy. Soc..

[39] Fraser DJ, Bernatchez L (2001). Adaptive evolutionary conservation: towards a unified concept for defining conservation units. Mol. Ecol..

[40] Simonov, E., Melnikov, D. & Orlov, N. Who is who in *Gloydius halys*-*intermedius* complex? New insights from extended geographical sampling and consequences for venom biochemistry research. *19th SEH European Symp*. (2017).

[41] Crisci, J. V., Katinas L. & Posadas, P. Historical biogeography: an introduction. (Harvard University Press, 2009).

[42] Matzke NJ (2014). Model selection in historical biogeography reveals that founder-event speciation is a crucial process in island clades. Syst. Biol..

[43] Matzke, N. J. Stochastic mapping under biogeographical models, http://phylo.wikidot.com/bio-geobears#stochastic_mapping (2015).

[44] Kayvanfar N, Aliabadian M, Niu X, Zhang Z, Liu Y (2017). Phylogeography of the Common Pheasant *Phasianus colchicus*. Ibis..

[45] Alaei Kakhki N (2018). Phylogeography of the *Oenanthe hispanica*–*pleschanka*–*cypriaca* complex (Aves, Muscicapidae: Saxicolinae): Diversification history of open‐habitat specialists based on climate niche models, genetic data, and morphometric data. J. Zool. Syst. Evol. Res..

[46] Kehl M (2009). Quaternary climate change in Iran—the state of knowledge. Erdkunde..

[47] Blanchet G, Sanlaville P, Traboulsi M (1997). Le Moyen-Orient de 20,000 ans BP à 6,000 ans BP Essai de reconstitution paléoclimatique. Paléorient..

[48] Ehlers, E. Iran: Grundzüge einer geographischen Landeskunde. (Broschiert, 1980).

[49] Lanfear R, Calcott B, Ho SY, Guindon S (2012). PartitionFinder: combined selection of partitioning schemes and substitution models for phylogenetic analyses. Mol. Biol. Evol..

[50] Yamani M (2002). The geomorphology of Alamkooh glaciers. Geogr. Res. Quart..

[51] Moghimi, E. Climatic Geomorphology cold and glacial territory. (University of Tehran Press, 2008).

[52] Moghimi, E. Geomorphology of Iran. (University of Tehran Press, 2010).

[53] Bobek H (1937). Die Rolle der Eiszeit in Nordwestiran. Z. Gletscherkunde..

[54] Schweizer G (1970). Der kuh-e-Sabalan (Nordwestiran). *Beiträge* zur *Gletscherkunde* und Glazialgeomorphologie vorderasiatischer Hochgebirge. Tubinger Geogr. Stud..

[55] Wright HE, Minneapolis J (1962). Pleistocene glaciation in Kurdistan. Eiszeit. Gegen..

[56] Fragner BG (1983). Iran. Grundzüge einer geographischen Landeskunde. Wiez. Z. Morgenlandes..

[57] Preu, C. Die quartäre Vergletscherung der inneren Zardeh-Kuh-Gruppe (Zardeh-Kuh-Massiv), Zagros/Iran. (Augsburger Geographische Hefte 4, 1984).

[58] Webb T, Bartlein P (1992). Global changes during the last 3 million years: climatic controls and biotic responses. Annu. Rev. Ecol. Syst..

[59] Dubey S, Zaitsev M, Cosson JF, Abdukadier A, Vogel P (2006). Pliocene and Pleistocene diversification and multiple refugia in a Eurasian shrew (*Crocidura suaveolens* group). Mol. Phylogenet. Evol..

[60] Tralau, H. The recent and fossil distribution of some boreal and arctic montane plants in Europe. (Almqvist & Wiksell, 1963).

[61] Leestmans R (2005). Le refuge caspiens et son importance en biogéographie. Linn. Belg..

[62] Akhani H, Djamali M, Ghorbanalizadeh A, Ramezani E (2010). Plant biodiversity of Hyrcanian relict forests, N Iran: an overview of the flora, vegetation, paleoecology and conservation. Pak. J. Bot..

[63] Leroy SA (2013). Holocene vegetation history and sea level changes in the SE corner of the Caspian Sea: relevance to SW Asia climate. Quat. Sci. Rev..

[64] Naderi G (2014). Mitochondrial evidence uncovers a refugium for the fat dormouse (*Glis glis* Linnaeus, 1766) in Hyrcanian forests of northern Iran. Mamm. Biol..

[65] Ashrafzadeh MR, Kaboli M, Naghavi MR (2016). Mitochondrial DNA analysis of Iranian brown bears (*Ursus arctos*) reveals new phylogeographic lineage. Mamm. Biol..

[66] Nazarizadeh M, Kaboli M, Rezaie HR, Harisini JI, Pasquet E (2016). Phylogenetic relationships of Eurasian Nuthatches (*Sitta europaea* Linnaeus, 1758) from the Alborz and Zagros Mountains, Iran. Zool. Middle East..

[67] Weir JT, Schluter D (2004). Ice sheets promote speciation in boreal birds. Proc. R. Soc. B..

[68] Amato GD (1991). Species hybridization and protection of endangered animals. Science..

[69] O’Brien SJ, Mayr E (1991). Bureaucratic mischief: Recognizing endangered species and subspecies. Science..

[70] Ryder OA (1986). Species conservation and systematics: the dilemma of subspecies. Trends Ecol. Evol..

[71] Moritz C (1994). Defining ‘evolutionarily significant units’ for conservation. Trends Ecol. Evol..

[72] Kapli P (2013). Molecular phylogeny and historical biogeography of the Anatolian lizard *Apathya* (Squamata, Lacertidae). Mol. Phylogenet. Evol..

[73] Saint KM, Austin CC, Donnellan SC, Hutchinson MN (1998). C-mos, a nuclear marker useful for squamate phylogenetic analysis. Mol. Phylogenet. Evol..

[74] Burbrink FT, Lawson R, Slowinski JB (2000). Mitochondrial DNA phylogeography of the polytypic North American rat snake (*Elaphe obsoleta*): a critique of the subspecies concept. Evolution..

[75] Arèvalo E, Davis SK, Sites JW (1994). Mitochondrial DNA sequence divergence and phylogenetic relationships among eight chromosome races of the *Sceloporus grammicus* complex (Phrynosomatidae) in central Mexico. Syst. Biol..

[76] Lawson R, Slowinski JB, Crother BI, Burbrink FT (2005). Phylogeny of the Colubroidea (Serpentes): new evidence from mitochondrial and nuclear genes. Mol. Phylogenet. Evol..

[77] Fenwick MA, Mansour YT, Franks S, Hardy K (2011). Identification and regulation of bone morphogenetic protein antagonists associated with preantral follicle development in the ovary. Endocrinology..

[78] Guo P (2012). Out of Asia: natricine snakes support the Cenozoic Beringian dispersal hypothesis. Mol. Phylogenet. Evol..

[79] Wu, Y. Species diversification of Asian pitvipers driven by the Pleistocene climatic oscillations. (unpublished manuscript). Bethesda (MD): National Library of Medicine (US), National Center for Biotechnology Information; [1988] – Accession No. JX661205 and JX661228, “*Gloydius saxatilis*” voucher QS002 cytochrome *b* (cyt *b*) gene, partial cds; mitochondrial, https://www.ncbi.nlm.nih.gov/nuccore/JX661205.

[80] Tamura K, Stecher G, Peterson D, Filipski A, Kumar S (2013). MEGA6: molecular evolutionary genetics analysis version 6.0. Mol. Biol. Evol..

[81] Librado P, Rozas J (2009). DnaSP v5: a software for comprehensive analysis of DNA polymorphism data. Bioinformatics..

[82] Ronquist F, Huelsenbeck JP (2003). MrBayes 3: Bayesian phylogenetic inference under mixed models. Bioinformatics..

[83] Rambaut A, Drummond AJ, Xie D, Baele G, Suchard MA (2018). Posterior summarisation in Bayesian phylogenetics using Tracer 1.7. Syst. Biol..

[84] Nguyen LT, Schmidt HA, von Haeseler A, Minh BQ (2014). IQ-TREE: a fast and effective stochastic algorithm for estimating maximum-likelihood phylogenies. Mol. Biol. Evol..

[85] Chernomor O, von Haeseler A, Minh BQ (2016). Terrace aware data structure for phylogenomic inference from supermatrices. Syst. Biol..

[86] Clement M, Posada D, Crandall KA (2000). TCS: a computer program to estimate gene genealogies. Mol. Ecol..

[87] Corander J, Sirén J, Arjas E (2008). Bayesian spatial modeling of genetic population structure. Computation. Stat..

[88] Cheng L, Connor TR, Sirén J, Aanensen DM, Corander J (2013). Hierarchical and spatially explicit clustering of DNA sequences with BAPS software. Mol. Biol. Evol..

[89] Excoffier L, Laval G, Schneider S (2005). Arlequin (version 3.0): an integrated software package for population genetics data analysis. Evol. Bioinform..

[90] Rannala B, Yang Z (2003). Bayes estimation of species divergence times and ancestral population sizes using DNA sequences from multiple loci. Genetics..

[91] Yang Z, Rannala B (2010). Bayesian species delimitation using multilocus sequence data. P. Natl. Acad. Sci. USA.

[92] Yang Z (2015). The BPP program for species tree estimation and species delimitation. Curr. Zool..

[93] Ziheng, Y. & Flouri, T. BP&P user manual, version 3.4 & 4.0, https://github.com/bpp/bpp (2018).

[94] Hudson RR, Kreitman M, Aguade M (1987). A test of neutral molecular evolution based on nucleotide data. Genetics..

[95] Bruen TC, Philippe H, Bryant D (2006). A simple and robust statistical test for detecting the presence of recombination. Genetics..

[96] Huson DH, Bryant D (2006). Application of phylogenetic networks in evolutionary studies. Mol. Biol. Evol..

[97] Zhang C, Zhang DX, Zhu T, Yang Z (2011). Evaluation of a Bayesian coalescent method of species delimitation. Syst. Biol..

[98] Leaché AD, Fujita MK (2010). Bayesian species delimitation in West African forest geckos (*Hemidactylus fasciatus*). Proc. R. Soc. B..

[99] Bagley JC (2015). Assessing species boundaries using multilocus species delimitation in a morphologically conserved group of neotropical freshwater fishes, the *Poecilia sphenops* species complex (Poeciliidae). PLOS ONE..

[100] Morcillo F, Ornelas-García CP, Alcaraz L, Matamoros WA, Doadrio I (2016). Phylogenetic relationships and evolutionary history of the Mesoamerican endemic freshwater fish family Profundulidae (Cyprinodontiformes: *Actinopterygii*). Mol. Phylogenet. Evol..

[101] Guo P (2016). Complex longitudinal diversification across South China and Vietnam in Stejneger’s pit viper, *Viridovipera stejnegeri* (Schmidt, 1925) (Reptilia: Serpentes: Viperidae). Mol. Ecol..

[102] Drummond AJ, Suchard MA, Xie D, Rambaut A (2012). Bayesian phylogenetics with BEAUti and the BEAST 1.7. Mol. Biol. Evol..

[103] Wüster, W. *et*. *al*. Origins and evolution of the South American pit viper fauna: evidence from mitochondrial DNA sequence analysis. *Biol*. *Vipers*. 111–128 (2002).

[104] Parmley D, Holman JA (2007). Earliest fossil record of a pigmy rattlesnake (Viperidae: *Sistrurus* Garman). J. Herpetol..

[105] Wüster W, Peppin L, Pook CE, Walker DE (2008). A nesting of vipers: phylogeny and historical biogeography of the Viperidae (Squamata: Serpentes). Mol. Phylogenet. Evol..

[106] Szyndlar Z, Rage JC (1999). Oldest fossil vipers (Serpentes: Viperidae) from the OldWorld. Kaupia..

[107] Head JJ, Mahlow K, Müller J (2016). Fossil calibration dates for molecular phylogenetic analysis of snakes 2: Caenophidia, Colubroidea, Elapoidea, Colubridae. Palaeontol. Electron..

[108] Drummond AJ, Rambaut A (2007). BEAST: Bayesian evolutionary analysis by sampling trees. BMC Evol. Biol..

[109] Brandley MC, Schmitz A, Reeder TW (2005). Partitioned Bayesian analyses, partition choice, and the phylogenetic relationships of scincid lizards. Syst. Biol..

[110] Matzke, N. J. BioGeoBEARS: BioGeography with Bayesian (and likelihood) evolutionary analysis in R Scripts. R package, version 0.2 (2013).

[111] Maddison, W. & Maddison, D. Mesquite: a modular system for evolutionary analysis. version 3.04 (2015).

[112] Lemmon AR, Lemmon EM (2008). A likelihood framework for estimating phylogeographic history on a continuous landscape. Syst. Biol..

[113] Cornuet JM (2014). DIYABCv2.0: a software to make approximate Bayesian computation inferences about population history using single nucleotide polymorphism, DNA sequence and microsatellite data. Bioinformatics..

[114] Sasaki, K. Ecology, behavior and conservation of the Japanese Mamushi snake, *Gloydius Blomhoffii*: variation in compromised and uncompromised populations. (Oklahoma State University, 2007).

[115] Yakovleva, I. D. Reptiles of Kirghizia. (Frunze, 1964).

[116] Beerli, P. Migrate documentation version 3.2.1. Tallahasee, United States: Florida State University (2012).

[117] Hijmans RJ, Cameron SE, Parra JL, Jones PG, Jarvis A (2005). Very high resolution interpolated climate surfaces for global land areas. Int. J. Climatol..

[118] IONIA. Globcover land cover (2009).

[119] R Core Team. R: A language and environment for statistical computing. R Foundation for Statistical Computing (2016).

