# Supplementary material for: Evolutionary history and postglacial colonization of an Asian pit viper (Gloydius halys caucasicus) into Transcaucasia revealed by phylogenetic and phylogeographic analyses
Source: Sci Rep. 2019 Feb 4;9:1224. doi: 10.1038/s41598-018-37558-8 (PMC6362119; doi:10.1038/s41598-018-37558-8)
Supplement: Supplementary file 1 — Supplementary information [file 41598_2018_37558_MOESM1_ESM.docx]

**TITLE PAGE**

**Evolutionary history and postglacial colonization of an Asian pit viper (*Gloydius halys caucasicus*) into Transcaucasia revealed by phylogenetic and phylogeographic analyses**

**Running title: Phylogeny and phylogeography of the Caucasian pit viper**

Atefeh Asadi, Claudine Montgelard, Masoud Nazarizadeh, Akram Moghaddasi, Faezeh Fatemizadeh, Evgeniy Simonov, Haji Gholi Kami, Mohammad Kaboli^*^

* Corresponding Author: [mkaboli@ut.ac.ir](mailto:mkaboli@ut.ac.ir)

**Supplementary information:**

**Figure S1**


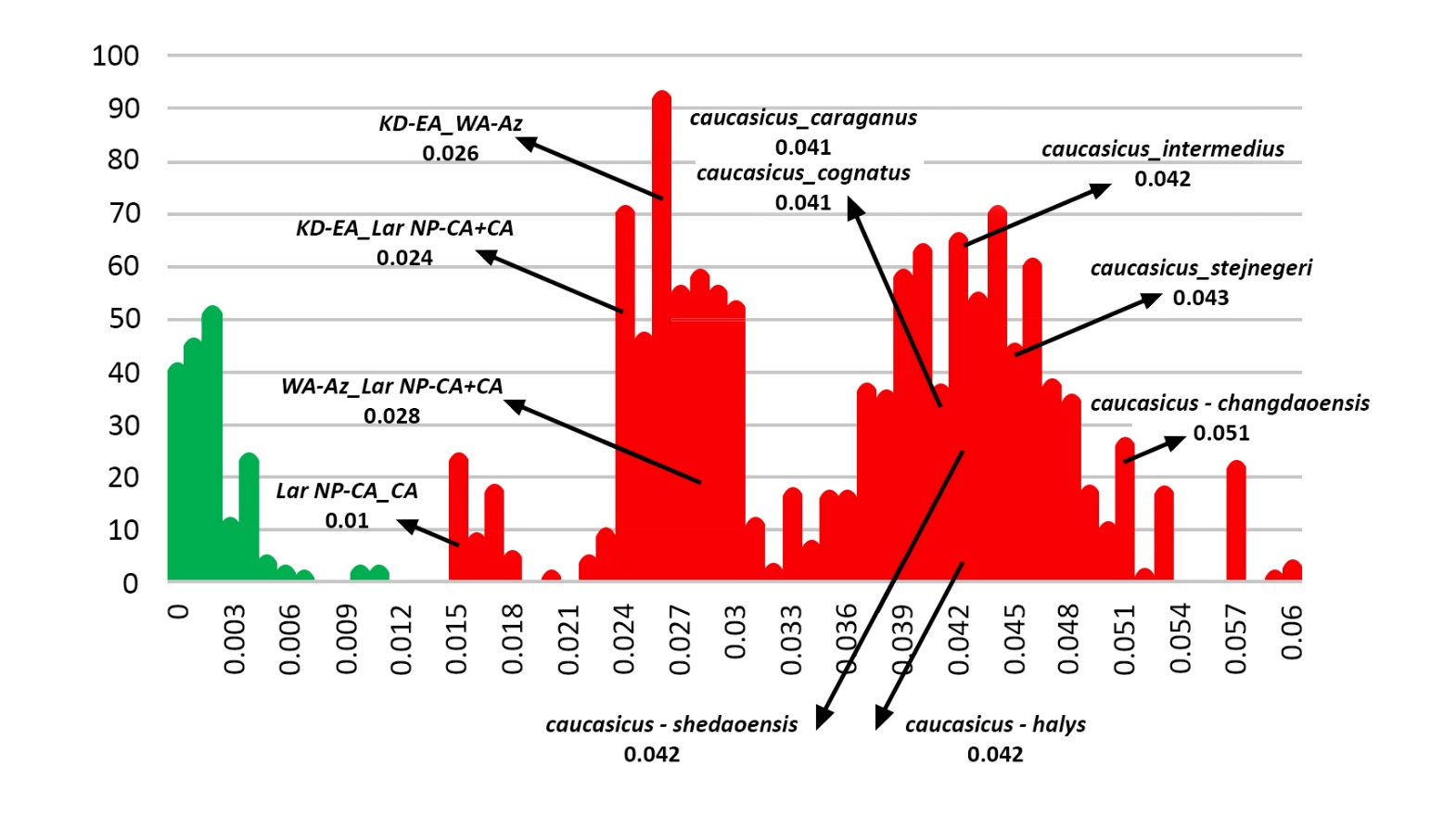


Figure S1. Barcoding gap in the *G. halys/G. intermedius* complex group using 54 sequences and 1551 bp of cyt *b*+ND4. Frequency distributions of intra-specific (green bars) and inter-specific (red bars) genetic divergences calculated using uncorrected p-distances with a total number of 1432 comparisons.

**Table S1**

Table S1. AMOVA among the four clusters of *G. caucasicus* in northern Iran and Azerbaijan using 53 sequences and 2370 bp of mtDNA + nDNA.

| Source of variation | Sum of  squares | Percentage of  variation | Fixation index | *P*-value |
| --- | --- | --- | --- | --- |
| Among lineages | 521.363 | 84.19 | FST = 0.84 | *P < 0.001* |
| Within lineages | 129.416 | 15.80 |  |  |
| Total | 650.779 |  |  |  |

**Table S2**

Table S2. Results of the BioGeoBEARS analysis using the concatenated mtDNA + nDNA dataset. LnL: log likelihood, K: parameters, d: dispersal, i: extinction, j: cladogenesis per-event weights, AIC: Akaike information criterion, and AICc: Akaike weight. Red bold line (DIVALIKE+J model) refers to the most probable pattern of dispersal, vicariance, and extinction for the *Gloydius* genus in the Palaearctic.

| **Scenario** | **Model** | **LnL** | **K** | **d** | **i** | **j** | **AIC** | **AICc-wt** |
| --- | --- | --- | --- | --- | --- | --- | --- | --- |
| **Scenario_HB_ S0** | DEC | -31.004 | 2 | 0.013201 | 0.0039 | 2.35E-08 | 74.40101 | 0.02090 |
|  | DEC+j | -32.4141 | 3 | 0.014247 | 0.0038 | 2.53E-08 | 75.40618 | 0.007289 |
|  | DIVALIKE | -30.2002 | 2 | 0.018086 | 0.0837 | 1.12E-12 | 68.42241 | 0.030893 |
|  | DIVALIKE+J | -31.7115 | 3 | 0.008126 | 0.0120 | 1.11E-12 | 64.86237 | 0.040912 |
|  | BAYAREALIKE | -60.0254 | 2 | 0.031741 | 0.0018 | 0.132161 | 132.0106 | 3.61E-13 |
|  | BAYAREALIKE+J | -47.7076 | 3 | 0.013125 | 0.0066 | 0.423169 | 97.61211 | 6.71E-08 |
|  |  |  |  |  |  |  |  |  |
| **Scenario_HB_ S1** | DEC | -31.3123 | 2 | 0.014410 | 0.0268 | 2.16E-08 | 72.12239 | 0.021871 |
|  | DEC+j | -36.2213 | 3 | 0.013219 | 0.0163 | 2.33E-08 | 74.50019 | 0.042121 |
|  | DIVALIKE | -31.2420 | 2 | 0.016213 | 0.0820 | 1.34E-12 | 65.00155 | 0.420131 |
|  | DIVALIKE+J | -28.7123 | 3 | 0.005121 | 0.0224 | 1.12E-12 | 66.86237 | 0.412221 |
|  | BAYAREALIKE | -63.234 | 2 | 0.031534 | 0.0133 | 0.452111 | 123.1145 | 3.65E-13 |
|  | BAYAREALIKE+J | -47.1241 | 3 | 0.013238 | 0.0134 | 0.422231 | 93.33129 | 6.60E-08 |
|  |  |  |  |  |  |  |  |  |
| **Scenario_HB_ S2** | DEC | -39.8107 | 2 | 0.012300 | 0.0232 | 2.98E-08 | 24.66331 | 0.011001 |
|  | DEC+j | -34.6351 | 3 | 0.012531 | 0.0340 | 2.43E-08 | 74.42617 | 0.020119 |
|  | DIVALIKE | -24.3818 | 2 | 0.027121 | 0.0831 | 1.00E-12 | 63.44141 | 0.566131 |
|  | **DIVALIKE+J** | **-18.8431** | **3** | **0.000125** | **0.051** | **1.00E-12** | **62.14381** | **0.604351** |
|  | BAYAREALIKE | -67.3501 | 2 | 0.031561 | 0.0117 | 0.403172 | 121.1930 | 3.51E-13 |
|  | BAYAREALIKE+J | -48.1113 | 3 | 0.013241 | 0.3113 | 0.311849 | 98.98042 | 6.19E-08 |
|  |  |  |  |  |  |  |  |  |
| **Scenario_HB_ S3** | DEC | -39.2007 | 2 | 0.010991 | 0.0095 | 2.10E-08 | 77.03511 | 0.011501 |
|  | DEC+j | -43.0011 | 3 | 0.012211 | 0000 | 2.05E-08 | 80.36891 | 0.006314 |
|  | DIVALIKE | -35.1176 | 2 | 0.017697 | 0.0421 | 1.03E-12 | 68.73131 | 0.219185 |
|  | DIVALIKE+J | -33.6078 | 3 | 0.008814 | 0.0302 | 1.02E-12 | 65.77237 | 0.308982 |
|  | BAYAREALIKE | -61.6180 | 2 | 0.036121 | 0.0172 | 0.434583 | 122.1889 | 3.02E-13 |
|  | BAYAREALIKE+J | -62.2101 | 3 | 0.015331 | 0.0131 | 0.423457 | 117.430 | 6.01E-08 |

**Table S3**

Table S3. Uncorrected p-distances for the concatenated cyt *b*+ND4 within the *G. halys* complex group. Values are presented as percentages, with the standard deviations in bold. Blue cells: average genetic distances among taxa of the *G. halys/G. intermedius* complex, grey cells: average genetic distance between the four Iranian clades of *G. h. caucasicus* and the other species of the complex, and green cells: average genetic distance among the four Iranian clades of *G. h. caucasicus.*

| EA - Az | CA | LarNP - CA | KD - EA | *G. h. halys* | *G. shedaoensis* | *G. changdaoensis* | *G. intermedius* | *G. caraganus* | *G. cognatus* | *G. stejnegeri* |  |
| --- | --- | --- | --- | --- | --- | --- | --- | --- | --- | --- | --- |
| **0.005** | **0.005** | **0.005** | **0.005** | **0.004** | **0.005** | **0.005** | **0.004** | **0.005** | **0.004** |  | *G. stejnegeri* |
| **0.004** | **0.004** | **0.004** | **0.004** | **0.004** | **0.005** | **0.005** | **0.004** | **0.004** |  | 4.0 | *G. cognatus* |
| **0.005** | **0.004** | **0.004** | **0.004** | **0.005** | **0.005** | **0.005** | **0.005** |  | 4.5 | 4.6 | *G. caraganus* |
| **0.005** | **0.005** | **0.005** | **0.005** | **0.004** | **0.003** | **0.005** |  | 3.7 | 3.7 | 3.9 | *G. intermedius* |
| **0.006** | **0.005** | **0.005** | **0.005** | **0.005** | **0.005** |  | 4.6 | 5.0 | 4.8 | 5.4 | *G. changdaoensis* |
| **0.005** | **0.005** | **0.005** | **0.005** | **0.004** |  | 4.5 | 1.0 | 3.9 | 3.7 | 4.0 | *G. shedaoensis* |
| **0.005** | **0.004** | **0.005** | **0.005** |  | 3.3 | 4.7 | 3.3 | 4.1 | 3.5 | 3.6 | *G. h. halys* |
| **0.004** | **0.003** | **0.004** |  | 3.6 | 3.7 | 4.6 | 3.7 | 3.8 | 3.8 | 4.2 | KD - EA |
| **0.004** | **0.003** |  | 2.5 | 4.0 | 4.4 | 5.1 | 4.3 | 4.3 | 4.2 | 4.3 | LarNP - CA |
| **0.004** |  | 1.5 | 2.3 | 4.0 | 4.3 | 5.3 | 4.3 | 4.1 | 4.2 | 4.5 | CA |
|  | 2.7 | 2.9 | 2.6 | 4.3 | 4.7 | 5.6 | 4.5 | 4.3 | 4.3 | 4.4 | WA - Az |

**Table S4**

Table S4. Sequence information from 53 individuals of *G. caucasicus*, representing 15 regions from northeastern to northwestern Iran and Azerbaijan along with 75 sequences obtained from GenBank.

|  | **Scientific name** | **Voucher**  **number** | **Locality** | **Accession number** | | | **Reference** |
| --- | --- | --- | --- | --- | --- | --- | --- |
|  |  |  |  | **cyt *b*** | **ND4** | **c-mos** |  |
| 1 | *Gloydius caucasicus* | NEZMUT_61 | Iran | MH378692 | MH378729 | MH891591 | present study |
| 2 | *Gloydius caucasicus* | NEZMUT_62 | Iran | MH378693 | MH378730 | MH891592 | present study |
| 3 | *Gloydius caucasicus* | NEZMUT_68 | Iran | MH378694 | MH378731 | MH891593 | present study |
| 4 | *Gloydius caucasicus* | NEZMUT_69 | Iran | MH378695 | MH378732 | MH891594 | present study |
| 5 | *Gloydius caucasicus* | NEZMUT_70 | Iran | MH378696 | MH378733 | MH891595 | present study |
| 6 | *Gloydius caucasicus* | NEZMUT_74 | Iran | MH378697 | MH378734 | MH891596 | present study |
| 7 | *Gloydius caucasicus* | NEZMUT_76 | Iran | MH378698 | MH378735 | MH891597 | present study |
| 8 | *Gloydius caucasicus* | NEZMUT_84 | Iran | MH378699 | MH378736 | MH891598 | present study |
| 9 | *Gloydius caucasicus* | NEZMUT_86 | Iran | MH378700 | MH378737 | MH891599 | present study |
| 10 | *Gloydius caucasicus* | NEZMUT_88 | Iran | MH378701 | MH378738 | MH891600 | present study |
| 11 | *Gloydius caucasicus* | NEZMUT_845 | Iran | MH378702 | MH378739 | MH891601 | present study |
| 12 | *Gloydius caucasicus* | NEZMUT_875 | Iran | MH378703 | MH378740 | MH891602 | present study |
| 13 | *Gloydius caucasicus* | NEZMUT_876 | Iran | MH378704 | MH378741 | MH891603 | present study |
| 14 | *Gloydius caucasicus* | NEZMUT_877 | Iran | MH378705 | MH378742 | MH891604 | present study |
| 15 | *Gloydius caucasicus* | NEZMUT_879 | Iran | MH378706 | MH378743 | MH891605 | present study |
| 16 | *Gloydius caucasicus* | NEZMUT_880 | Iran | MH378707 | MH378744 | MH891606 | present study |
| 17 | *Gloydius caucasicus* | NEZMUT_926 | Iran | MH378708 | MH378745 | MH891607 | present study |
| 18 | *Gloydius caucasicus* | NEZMUT_973 | Iran | MH378709 | MH378746 | MH891608 | present study |
| 19 | *Gloydius caucasicus* | NEZMUT_1059 | Iran | MH378710 | MH378747 | MH891609 | present study |
| 20 | *Gloydius caucasicus* | NEZMUT_1060 | Iran | MH378711 | MH378748 | MH891610 | present study |
| 21 | *Gloydius caucasicus* | NEZMUT_1061 | Iran | MH378712 | MH378749 | MH891611 | present study |
| 22 | *Gloydius caucasicus* | NEZMUT_1063 | Iran | MH378713 | MH378750 | MH891612 | present study |
| 23 | *Gloydius caucasicus* | NEZMUT_1198 | Iran | MH378714 | MH378751 |  | present study |
| 24 | *Gloydius caucasicus* | NEZMUT_1238 | Iran | MH378715 | MH378752 |  | present study |
| 25 | *Gloydius caucasicus* | NEZMUT_1241 | Iran | MH378716 | MH378753 |  | present study |
| 26 | *Gloydius caucasicus* | NEZMUT_1243 | Iran | MH378717 | MH378754 |  | present study |
| 27 | *Gloydius caucasicus* | NEZMUT_1255 | Iran | MH378718 | MH378755 |  | present study |
| 28 | *Gloydius caucasicus* | NEZMUT_1260 | Iran | MH378719 | MH378756 |  | present study |
| 29 | *Gloydius caucasicus* | NEZMUT_1277 | Iran | MH378720 | MH378757 |  | present study |
| 30 | *Gloydius caucasicus* | NEZMUT_1278 | Iran | MH378721 | MH378758 |  | present study |
| 31 | *Gloydius caucasicus* | NEZMUT_1281 | Iran | MH378722 | MH378759 |  | present study |
| 32 | *Gloydius caucasicus* | NEZMUT_1282 | Iran | MH378723 | MH378760 |  | present study |
| 33 | *Gloydius caucasicus* | NEZMUT_1283 | Iran | MH378724 | MH378761 |  | present study |
| 34 | *Gloydius caucasicus* | NEZMUT_1293 | Iran | MH378725 | MH378762 |  | present study |
| 35 | *Gloydius caucasicus* | NEZMUT_1294 | Iran | MH378726 | MH378763 |  | present study |
| 36 | *Gloydius caucasicus* | NEZMUT_1297 | Iran | MH378727 | MH378764 |  | present study |
| 37 | *Gloydius caucasicus* | NEZMUT_1299 | Iran | MH378728 | MH378765 |  | present study |
| 38 | *Gloydius caucasicus* | G68 | Azerbaijan | MH370352 | MH370354 |  | present study |
| 39 | *Gloydius caucasicus* | G69 | Azerbaijan | MH370353 | MH370355 |  | present study |
| 40 | *Gloydius caucasicus* | GH26 | Azerbaijan | MH370350 | MH370357 |  | present study |
| 41 | *Gloydius caucasicus* | GH27 | Azerbaijan | MH370351 | MH370356 |  | present study |
| 42 | *Gloydius caucasicus* | 1031 | Iran | KX855938 |  |  | Rastegar- pouyani *et al.,* 2018 |
| 43 | *Gloydius caucasicus* | 3517 | Iran | KX855939 |  |  | Rastegar- pouyani *et al.,* 2018 |
| 44 | *Gloydius caucasicus* | 3507 | Iran | KX855940 |  |  | Rastegar- pouyani *et al.,* 2018 |
| 45 | *Gloydius caucasicus* | 3514 | Iran | KX855941 |  |  | Rastegar- pouyani *et al.,* 2018 |
| 46 | *Gloydius caucasicus* | 3512 | Iran | KX855942 |  |  | Rastegar- pouyani *et al.,* 2018 |
| 47 | *Gloydius caucasicus* | 3511 | Iran | KX855943 |  |  | Rastegar- pouyani *et al.,* 2018 |
| 48 | *Gloydius caucasicus* | 3509 | Iran | KX855944 |  |  | Rastegar- pouyani *et al.,* 2018 |
| 49 | *Gloydius caucasicus* | 3500 | Iran | KX855950 |  |  | Rastegar- pouyani *et al.,* 2018 |
| 50 | *Gloydius caucasicus* | 3501 | Iran | KX855949 |  |  | Rastegar- pouyani *et al.,* 2018 |
| 51 | *Gloydius caucasicus* | 3502 | Iran | KX855948 |  |  | Rastegar- pouyani *et al.,* 2018 |
| 52 | *Gloydius caucasicus* | 3503 | Iran | KX855947 |  |  | Rastegar- pouyani *et al.,* 2018 |
| 53 | *Gloydius caucasicus* | 3504 | Iran | KX855946 |  |  | Rastegar- pouyani *et al.,* 2018 |
| 54 | *Gloydius stejnegeri* | JSSD1409S3 | Tongchuan, Shaanxi | KX063817 | KX063790 |  | Shi *et al.,* 2017 |
| 55 | *Gloydius stejnegeri* | JSSD1508S4 | Linfen, Shanxi | [KX063818](https://www.ncbi.nlm.nih.gov/nucleotide/KX063818?report=genbank&log$=nuclalign&blast_rank=4&RID=8KEH2YKP015) | KX063791 |  | Shi *et al.,* 2017 |
| 56 | *Gloydius stejnegeri* | SYNU1510134 |  | KX063816 | KX063789 |  | Shi *et al.,* 2017 |
| 57 | *Gloydius stejnegeri* | SYNU1510145 | Mentougou, Beijing | [KX063815](https://www.ncbi.nlm.nih.gov/nucleotide/KX063815?report=genbank&log$=nuclalign&blast_rank=2&RID=8KEH2YKP015) | KY040570 |  | Shi *et al.,* 2016 |
| 58 | *Gloydius stejnegeri* | JS151054 | Mentougou, Beijing | KY040625 | KY040646 |  | Shi *et al.,* 2017 |
| 59 | *Gloydius cognatus* | BYEB1501IA |  | KX063812 | KX063785 |  | Shi *et al.,* 2016 |
| 60 | *Gloydius cognatus* | JS130947 |  | KY040643 | KY040642 |  | Shi *et al.,* 2016 |
| 61 | *Gloydius cognatus* | CIB93145 |  | KX063811 | KX063784 |  | Shi *et al.,* 2016 |
| 62 | *Gloydius cognatus* | JS15100005 |  | KX063814 | KX063787 |  | Shi *et al.,* 2017 |
| 63 | *Gloydius cognatus* | CIBQY224 | Zoige, Sichuan | KY040619 | KY040640 |  | Shi *et al.,* 2017 |
| 64 | *Gloydius cognatus* | JSSD13109I3 | Sonit Right Banner, Inner Mongolia | KY040621 | KY040642 |  | Shi *et al.,* 2017 |
| 65 | *Gloydius cognatus* | JS131147 | Yinchuan, Ningxia | KY040622 | KY040643 |  | Shi *et al.,* 2017 |
| 66 | *Gloydius cognatus* | JSSD1504N6 | Wuzhong, Ningxia | KX063809 | KX063782 |  | Shi *et al.,* 2017 |
| 67 | *Gloydius halys halys* | DLHSKG50 |  | KX063807 | KX063780 |  | Shi *et al.,* 2016 |
| 68 | *Gloydius halys halys* | SYNU1510151 |  | KX063800 | KX063773 |  | Shi *et al.,* 2016 |
| 69 | *Gloydius halys halys* | JSSD140M1 |  | KX063804 | KX063777 |  | Shi *et al.,* 2016 |
| 70 | *Gloydius halys halys* | DLG11 |  | KX063805 | KX063778 |  | Shi *et al.,* 2016 |
| 71 | *Gloydius halys halys* | DLG12 |  | KX063806 | KX063779 |  | Shi *et al.,* 2016 |
| 72 | *Gloydius halys halys* | SYNU1301908 | Lingyuan, Liaoning | KX063802 | KX063775 |  | Shi *et al.,* 2017 |
| 73 | *Gloydius halys halys* | JSSD1508X3 | Xilinhot, Inner | KX063803 | KX063776 |  | Shi *et al.,* 2017 |
| 74 | *Gloydius halys halys* | JS1407H9 | Greater Hinggan Mts | KY040618 | KY040639 |  | Shi *et al.,* 2017 |
| 75 | *Gloydius rickmersi* | MHNG 2752.69 | Kyrgyzstan |  | KM078592 |  | Fenwick *et al.,* 2011 |
| 76 | *Gloydius rickmersi* | MHNG 2752.70 | Kyrgyzstan |  | KM096379 |  | Wagner *et al.,* 2016 |
| 77 | *Gloydius caraganus* | ISEA R290 |  |  | KM078594 |  | Wagner *et al.,* 2016 |
| 78 | *Gloydius caraganus* | CR1 |  | MF490455 | MF490453 |  | Shi *et al.,* 2017 |
| 79 | *Gloydius caraganus* | CR2 |  | MF490456 | MF490454 |  | Shi *et al.,* 2017 |
| 80 | *Gloydius changdaoensis* | JSSD1408Z1 | Lianyungang, Jiangsu | KX063821 | KX063794 |  | Shi *et al.,* 2017 |
| 81 | *Gloydius changdaoensis* | JSSD1510C1 | Changdao, Shandong | KX063823 | KX063796 |  | Shi *et al.,* 2017 |
| 82 | *Gloydius shedaoensis* | GP1100 |  | JQ687498 | JQ687479 | JQ687517 | Xu *et al.,* 2012 |
| 83 | *Gloydius shedaoensis* | GP1116 |  | JQ687500 | JQ687481 | JQ687519 | Xu *et al.,* 2012 |
| 84 | *Gloydius shedaoensis* | GP1110 |  | JQ687499 | JQ687480 | JQ687518 | Xu *et al.,* 2012 |
| 85 | *Gloydius shedaoensis* | HS110682 |  | KF997910 | KF997969 |  | Huang, 2013 |
| 86 | *Gloydius shedaoensis* | HS11062 |  | KF997911 | KF997971 |  | Huang, 2013 |
| 87 | *Gloydius shedaoensis* | HS11067 |  | KF997912 | KF997970 |  | Huang, 2013 |
| 88 | *Gloydius shedaoensis* | JSSD1510C1 | Lvshun, Liaoning | KX063819 | KX063792 |  | Shi *et al.,* 2017 |
| 89 | *Gloydius intermedius* | JS150622 | Zhuanghe, Liaoning | KY040617 | KY040638 |  | Shi *et al.,* 2017 |
| 90 | *Gloydius intermedius* | QS002 | Anshan, Liaoning | JX661205 | JX661228 |  | Wu, 2015 |
| 91 | *Gloydius intermedius* | GP1328 | Ji'an, Jilin, China | JQ687502 | JQ687483 | JQ687521 | Xu *et al.,* 2012 |
| 92 | *Gloydius intermedius* | GP191 | Hengren, Liaonling | JQ687489 | JQ68740 | JQ687508 | Xu *et al.,* 2012 |
| 93 | *Gloydius brevicaudus* | GP628 | Kuandian, Liaoning | JQ687495 | JQ687476 | JQ687514 | Xu *et al.,* 2012 |
| 94 | *Gloydius ussuriensis* | GP1326 |  | JQ687501 | JQ687482 | JQ687520 | Xu *et al.,* 2012 |
| 95 | *Gloydius liupanensis* | GP215 | Ningxia, China | JQ687493 | JQ687474 | JQ687512 | Xu *et al.,* 2012 |
| 96 | *Gloydius blomhoffi* | B524 | Japan | AY352751 | AY352814 |  | Malhotra *et al.,* 2003 |
| 97 | *Gloydius tsushimaensis* | Ts1 | Japan | JN870203 | JN870211 |  | Fenwick *et al.,* 2011 |
| 98 | *Gloydius brevicaudus* | DW004 |  | JX661198 | JX661225 |  | Guo *et al.,* 2011 |
| 99 | *Gloydius brevicaudu* | DW005 |  | JX661199 | JX661226 |  | Wüster *et al.,* 2008 |
| 100 | *Gloydius brevicaudus* | GP52 |  |  |  | JQ687505 | Xu et al., 2012 |
| 101 | *Gloydius brevicaudus* | GP1027 |  |  |  | JQ687515 | Xu et al., 2012 |
| 102 | *Gloydius brevicaudus* | GP1099 |  |  |  | JQ687516 | Xu et al., 2012 |
| 103 | *Gloydius brevicaudus* | GP1418 |  |  |  | JQ687523 | Xu et al., 2012 |
| 104 | *Gloydius ussuriensis* | GP1331 |  |  |  | JQ687522 | Xu et al., 2012 |
| 105 | *Gloydius* *liupanensis* | GP206 |  |  |  | JQ687510 | Xu et al., 2012 |
| 106 | *Gloydius* *liupanensis* | GP215 |  |  |  | JQ687511 | Xu et al., 2012 |
| 107 | *Gloydius intermedius* | GP465 |  |  |  | JQ687506 | Xu et al., 2012 |
| 108 | *Gloydius intermedius* | GP188 |  |  |  | JQ687513 | Xu et al., 2012 |
| 109 | *Gloydius intermedius* | GP188 |  |  |  | JQ687507 | Xu et al., 2012 |
| 110 | *Trimeresurus sichuanensis* |  | Sichuan | KT2668 | KT2668 |  | Zhu *et al.,* 2015 |
| 111 | *Deinagkistrodon acutus* |  | Fujian | DQ343647 | DQ343647 |  | Yan *et al.,* 2008 |
| 112 | *Sistrurus catenatus* |  |  | AY223610 | AY223648 |  | Wüster *et al.,* 2008 |
| 113 | *Sistrurus miliarius* |  |  | AY223611 | U41889 |  | Wüster *et al.,* 2008 |
| 114 | *Crotalus adamanteus* |  |  | AY223605 | U41880 |  | Wüster *et al.,* 2008 |
| 115 | *Crotalus ravus* |  |  | AY223609 | AY223647 |  | Wüster *et al.,* 2008 |
| 116 | *Crotalus simus* |  |  | EU624302 | AY704885 |  | Wüster *et al.,* 2008 |
| 117 | *Crotalus tigris* |  |  | AY223606 | AF156574 |  | Wüster *et al.,* 2008 |
| 118 | *Porthidium arcosae* |  |  | AF292575 | AF292613 |  | Wüster *et al.,* 2008 |
| 119 | *Porthidium lansbergii* |  |  | AF713375 | AF393623 |  | Wüster *et al.,* 2008 |
| 120 | *Porthidium nasutum* |  |  | AF223579 | U41887 |  | Wüster *et al.,* 2008 |
| 121 | *Porthidium ophryomegas* |  |  | AY713375 | AF393623 |  | Wüster *et al.,* 2008 |
| 122 | *Vipera berus* |  |  | DQ186079 |  |  | Wüster *et al.,* 2008 |
| 123 | *Vipera ammodytes* |  |  | DQ186513 | EU624232 |  | Wüster *et al.,* 2008 |
| 124 | *Daboia mauritanica* |  |  | NC036956 |  |  | Wüster *et al.,* 2008 |
| 125 | *Macrovipera lebetina* |  |  | KJ415301 |  |  | Wüster *et al.,* 2008 |
| 126 | *Macrovipera schweizeri* |  |  | AJ275715 |  |  | Wüster *et al.,* 2008 |
| 127 | *Montivipera albizona* |  |  | KX168728 |  |  | Wüster *et al.,* 2008 |
| 128 | *Montivipe xanthina* |  |  | KX168811 |  |  | Wüster *et al.,* 2008 |

**Table S5**

Table S5. List of primers used for the two mitochondrial genes (cyt *b* and ND4) and one nuclear gene (c-mos) amplification and PCR protocols.

| Primer | Gene | Sequence (5’-3’) | Reference |
| --- | --- | --- | --- |
| L14910 | cyt *b* | GAC CTG TGA TMT GAA AAC CAY CGT TGT | Burbrink *et al*., 2000 |
| H16064 | cyt *b* | CTT TGG TTT ACA AGA ACA ATG CTT TA |  |
| ND4 | ND4 | CAC CTA TGA CTA CCA AAA GCT CAT GTA GAA GC | Arevalo *et al*., 1994 |
| Leu | ND4 | CAT TAC TTT TAC TTG GAT TTG CAC CA |  |
| S77  S78 | c-mos  c-mos | CAT GGA CTG GGA TCA GTT ATG  CCT TGG GTG TGA TTT TCT CAC CT | Lawson *et al.,* 2005 |

PCR conditions for cyt *b*: For amplifying target genes, PCR was performed in a 45 µL final volume, including 3-6 µL of DNA template, 10X PCR buffer (Qiagen), 25 mм MgCl2, 0.25 mм of dNTP, 10 µм of each primer and 1 unit of Taq polymerase (Qiagen). Amplification conditions consisted of 34 cycles as follows: denaturation for 45 seconds at 94°C, annealing for 60 s at 50°C for cyt *b* and at 52°C for ND4, and extension for 3 minutes at 72°C.

PCR conditions for ND4: Each 50 µl polymerase chain reaction (Qiagen) contained ±50 ng of total DNA, 350 mM of each dNTP, 0.5 pmol of each primer, 2 units of DNA polymerase and a final concentration of 1.5 mM MgCl2. The PCR profile included 2.5 minutes preheating at 94°C and 35 cycles of 30 seconds at 94°C, 30 seconds at 55°C and 45 seconds at 72°C. PCR cycles were followed by 10 minutes of extension at 72°C.

PCR conditions for c-mos: PCR amplification was carried out in a 25μl reaction using PCR master mix (Ampliqon, Denmark) containing Tris-HCl (pH 8.5), (NH_4_)_2_SO_4_, 3 mM MgCl_2_, 0.2 % Tween®20, 0.4 mM of each dNTP, and 0.2 units of Taq DNA Polymerase (Ampliqon). A PCR thermal programing with three different stages in a range of 52–60 °C was used in which the temperature was gradually reduced. Cycling included an initial denaturation step of 5 minutes at 95 °C, 35 cycles at 94 °C for 30s, followed by annealing at 54.5 °C for 40s, 72 °C for 1 min (extension) and 72 °C for 5 min (final extension).
